# Spatial and Sequential Topological Analysis of Molecular Dynamics Simulations of IgG1 Fc Domains

**DOI:** 10.1021/acs.jctc.5c00161

**Published:** 2025-04-22

**Authors:** Melinda Kleczynski, Christina Bergonzo, Anthony J. Kearsley

**Affiliations:** † National Institute of Standards and Technology, Gaithersburg, Maryland 20899, United States; ‡ Institute for Bioscience and Biotechnology Research, Rockville, Maryland 20850, United States

## Abstract

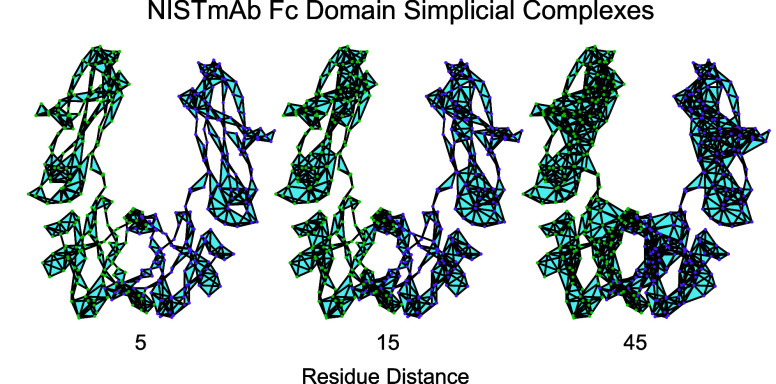

Monoclonal antibodies are utilized in a wide range of biomedical applications. The NIST monoclonal antibody is a resource for developing analysis methods for monoclonal antibody based biopharmaceutical platforms. Techniques from topological data analysis quantify structural features such as loops and tunnels which are not easily measured by classical data analysis methods. In this paper, we introduce the Gaussian CROCKER column differences (GCCD) matrix, which augments standard topological data analysis summaries with biological sequence information. We use GCCD matrices to successfully differentiate between glycosylated and aglycosylated conformations from molecular dynamics simulations of the NIST monoclonal antibody Fc domain. We are optimistic that other researchers will be able to utilize GCCD matrices to quantify multiscale spatial and sequential features.

## 1 Introduction

Monoclonal antibodies are approved for use in a wide range of medical applications^1^ including treatment of multiple types of cancers^2,3^ and autoimmune diseases.^3^ Consequently, monoclonal antibodies have substantial commercial and economic impact.^4,5^ Analysis of monoclonal antibodies is of increasing importance. The National Institute of Standards and Technology (NIST) maintains numerous reference materials, including the NIST monoclonal antibody (NISTmAb), Reference Material (RM) 8671.^6^ The NISTmAb facilitates development and evaluation of analysis methods for monoclonal antibody based biopharmaceutical platforms.

**Figure 1:**
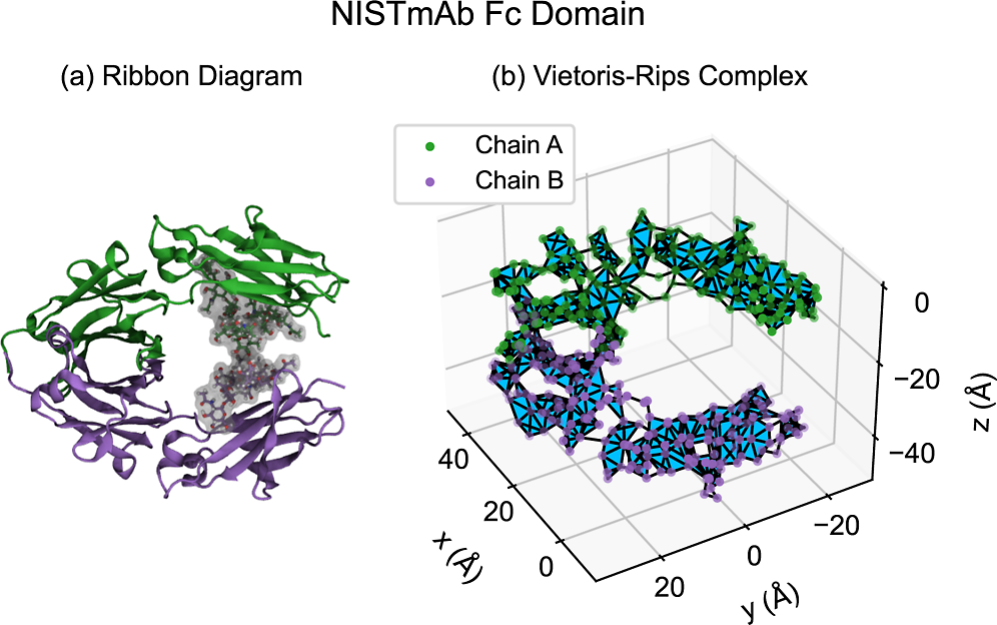
NISTmAb Fc domain contains two amino acid chains (shown in green and purple). (a) The Fc domain, shown here as a ribbon diagram,^11^ can be accompanied by glycans (gray shading). (b) The Fc domain can be represented as a Vietoris–Rips complex using α carbon atoms as vertices. Simplices connect sets of vertices corresponding to α carbons which are spatially close to each other.

Many factors influence antibody function, including glycan (polysaccharide) presence and composition.^7^Figure 1a shows the NISTmAb crystallizable fragment (Fc) domain with glycans present, in other words in a glycosylated form. One way to explore the effects of glycosylation is to simulate antibody conformations with and without glycans. By modeling atomic particle motion, molecular dynamics simulations generate a range of possible configurations of molecules at atomic resolution, serving as a useful complement to experimental data.^8,9^ Molecular dynamics simulations of glycosylated and aglycosylated Fc domains are known to be similar with respect to standard descriptors.^10^

Topological data analysis (TDA) techniques can be used to detect and quantify multiscale structure of data sets, generating new features compared to classical analysis methods.^12^ TDA has been successfully deployed for a wide range of applications, particularly in biology^13^ and biomedicine.^14^ Biomolecular topology is a growing field which applies techniques from topological data analysis and related disciplines to the study of biomolecules.^15^ We utilize TDA to analyze glycosylated and aglycosylated molecular dynamics simulations of the NISTmAb. We produce matrix summaries of topological structure which are well-suited for subsequent classification, clustering, or machine learning tasks, either in place of or in addition to standard descriptors.

The branch of TDA employed in this work is persistent homology^16^ (persistent homology is a mathematical term which is distinct from the use of homology to indicate sequence similarity^17^ or common ancestry^18^). A common framework for applying persistent homology is analysis of a finite set of data points in 2-dimensional or 3-dimensional space; we call this a point cloud. In typical persistent homology of point cloud data, the points are unordered. An example would be points indicating spatial positions of some collection of organisms, where no individual organism is ranked above or below any other individual. For biomolecules, points may be equipped with an order or partial order. In molecular dynamics simulations of the NISTmAb, the α carbon atoms correspond to a set of sequences, one for each chain of the protein dimer.

For all analysis in the current work, the point clouds consist of α carbon atoms. These α carbons have positions in 3-dimensional space, like points in a typical point cloud. Unlike typical point clouds, the α carbons of an Fc domain also belong to one of two chains, and the corresponding residues have sequential positions within their chain.

Existing approaches which can incorporate sequence information in TDA of biomolecules include optimal cycle representatives and slicing. For optimal cycle representatives, the definition of an optimal cycle can utilize the sequential structure.^19^ We discuss cycle representatives and associated challenges in Section 2.4. Slicing techniques involve choosing a small portion of a protein and then producing persistent homology summaries while progressively including more of the protein; for example, one can add α carbons in sequential order by moving along an α helix.^20^ Slicing approaches have been used to reveal topological signatures of local structures such as α helices and β sheets.^20^

We utilize Euclidean distances between the 3-dimensional spatial coordinates of α carbon atoms, and differences between the sequence positions of the corresponding residues. Spatial distances are continuous, while residue distances have discrete integer values. We require a topological summary which tracks persistent homology with respect to a continuous spatial distance parameter and a discrete residue distance parameter. “Contour Realization Of Computed k-dimensional hole Evolution in the Rips complex” (CROCKER) plots^21^ are a good starting point for our purposes. CROCKER plots and matrices have been used for visualization and analysis of discrete time, continuous space models of collective motion.^21,22^ Similar approaches involving repeatedly computing 1-parameter persistent homology have been used for applications including protein folding/unfolding^23^ and drug discovery.^24^ In this paper we introduce the Gaussian CROCKER column differences (GCCD) matrix, a variation of a CROCKER matrix which leverages sequence information in the form of residue distances.

We successfully use GCCD matrices to classify simulated glycosylated versus aglycosylated conformations of the NISTmAb Fc domain. Using a simple classification method, GCCD matrices yield better performance than comparable vectorized topological summaries which only incorporate spatial distances. The GCCD matrix provides a framework for summarizing topological structure with respect to spatial and sequential distances, and has potential applicability to other biomolecules.

## 2 Topological Data Analysis Background

In this section we provide TDA background relevant to this paper. More comprehensive discussions of topological data analysis can be found in introductory texts.^25,26^ We lay the groundwork for our introduction of GCCD matrices by reviewing the existing techniques of persistent homology, Betti curves and vectors, and CROCKER plots and matrices. We also review cycle representatives, which we use for visualization. Once GCCD matrices have been obtained, we use the established technique of *k*-nearest neighbors classification to differentiate glycosylated versus aglycosylated conformations. We also use multidimensional scaling (MDS) for visualization.

### 2.1 Persistent Homology

**Figure 2:**
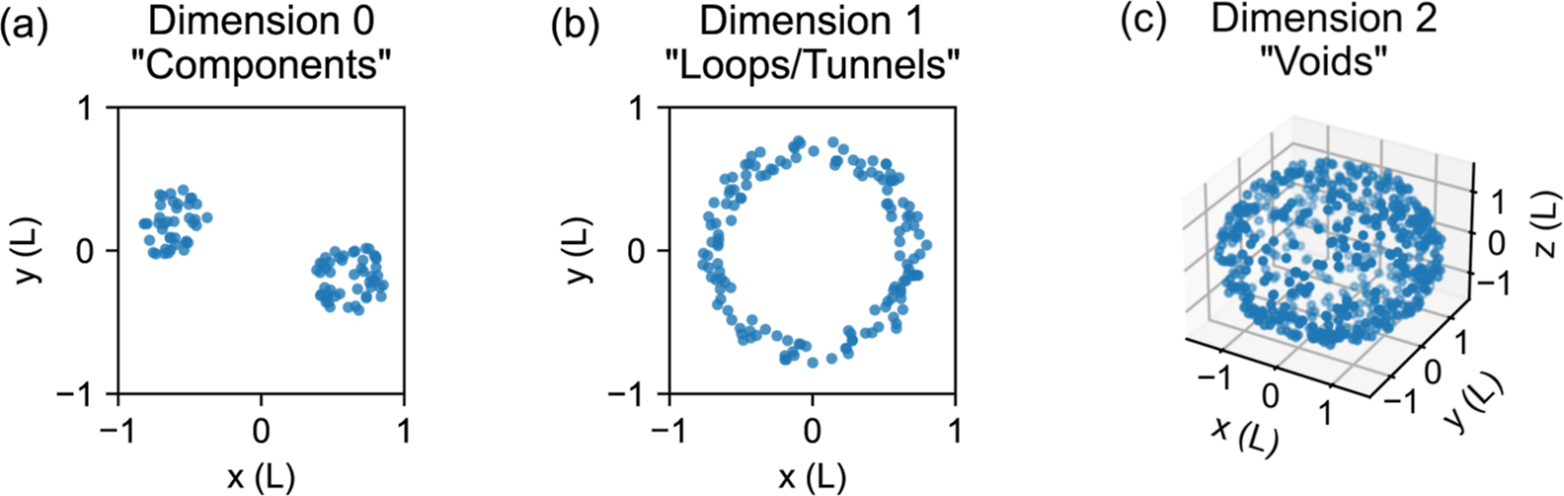
Different types of empty spaces in point cloud data are detectable by persistent homology in different dimensions. L indicates an arbitrary unit of length. (a) Dimension 0 persistent homology detects separations between groups of data points. Connecting nearby points creates connected components associated with these groups. Dimension 0 persistent homology corresponds to a hierarchical clustering of the data set. (b) Dimension 1 persistent homology detects data points bordering empty spaces, where connecting nearby points creates loops or tunnels. (c) Dimension 2 persistent homology detects data points forming enclosed volumes, where connecting nearby points creates voids.

Persistent homology detects different types of empty spaces enclosed by data points, as shown in Figure 2. In the context of proteins, these structures include components, tunnels, and voids.^27,28^ Dimension 0 persistent homology detects gaps between groups of data points, as shown in Figure 2a. These groups of data points form different connected components when connections are added between nearby points. For point clouds consisting of α carbon positions, dimension 0 persistent homology may not contain interesting large-scale structure, because each α carbon is spatially close to the α carbons of the adjacent residues from the amino acid chain. Dimension 1 persistent homology detects data points bordering empty spaces in the form of loops or tunnels, as shown in Figure 2b. Loops/tunnels may be flattened like a hoop or elongated like a drinking straw; typical persistent homology pipelines do not distinguish between these. Dimension 2 persistent homology detects data points arranged in shells surrounding empty spaces in the form of enclosed volumes or voids, as shown in Figure 2c. All of these structures may be irregularly shaped. We focus on dimension 1 persistent homology in the current work, but other dimensions are of interest for other applications. For example, dimension 2 persistent homology is useful for analyzing molecular dynamics simulations of membrane fusion.^29^

**Figure 3:**
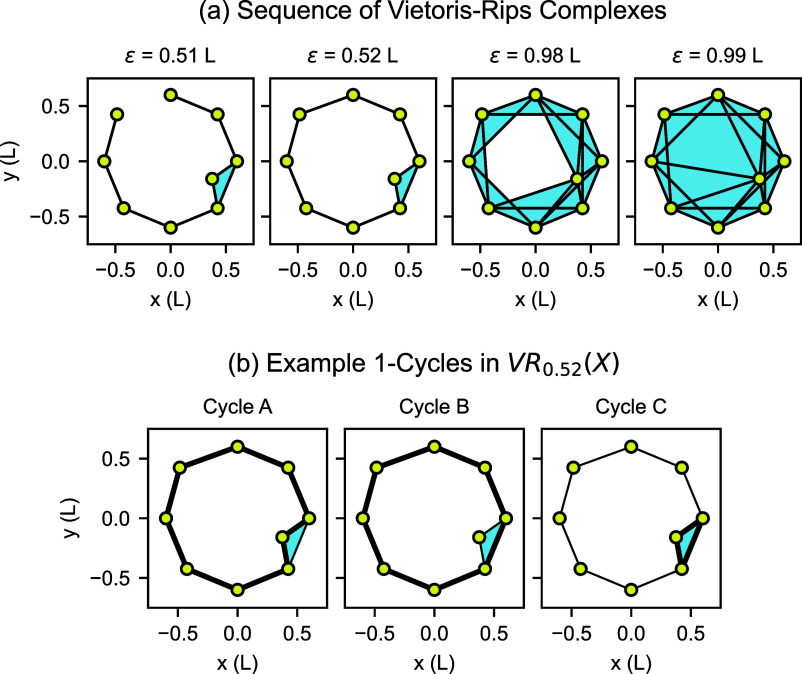
Vietoris–Rips complexes are formed by connecting sets of points with pairwise distances at most ε. (a) A “loop” or “tunnel” appears at ε = 0.52 L and disappears at ε = 0.99 L. (b) Three examples of 1-cycles are marked by bold edges. Cycles A and B enclose the empty region at the center of the tunnel, while cycle C is the boundary of a 2-simplex. Cycle A and cycle B would both be valid cycle representatives corresponding to the tunnel structure of this data set.

We begin with a small example of persistent homology in dimension 1, with selected simplicial complexes shown in Figure 3a. These are part of a sequence of simplicial complexes, called a filtration, corresponding to increasing values of the parameter ε. Our example data set *X* consists of the points plotted in gold. In this data set, nearby points can be connected to form a tunnel. We accomplish this by constructing a sequence of Vietoris–Rips complexes. Each Vietoris–Rips complex, denoted VR_ε_(*X*), has vertices corresponding to the points in the data set *X* and simplices connecting all sets of points with pairwise distance at most ε. For dimension 1 persistent homology, we consider 1-simplices, which connect pairs of vertices and are drawn as edges, and 2-simplices, which connect sets of three vertices and are drawn as triangles.

A Vietoris–Rips complex is one way to represent point cloud data as a mathematical object called a simplicial complex,^30^ and is the type of simplicial complex typically used in CROCKER plots and matrices.^21^Figure 1b shows the NISTmAb Fc domain represented as a Vietoris–Rips complex, with vertices at the positions of the α carbon atoms. Figure 3a shows four Vietoris–Rips complexes constructed from our example data set *X* for different values of ε. For now, we focus on the second Vietoris–Rips complex, VR_0.52_(*X*).

Figure 3b shows examples of collections of 1-simplices which form 1-cycles. Note that in topological data analysis, we are permitted to add two cycles to obtain a new cycle; this may result in cycles that are more complex than the ones we have shown here. In general, one could consider *k*-cycles for other values of *k*, but in this work we focus on 1-cycles, which we may refer to simply as cycles.

In Figure 3b, cycles A and B enclose the empty region at the center of the tunnel. It would be reasonable to mark the tunnel location using either cycle A or cycle B, but cycle C does not enclose the empty space and does not reflect the tunnel structure. A key strategy in topological data analysis is to group cycles together in classes. We place cycles A and B in the same class, while cycle C is placed in the zero class. Note that cycles A and B differ by the boundary of a 2-simplex, while cycle C is the boundary of a 2-simplex. In general, we place cycles in the same class if they differ by boundaries, and we place cycles that are boundaries in the zero class. In dimension 1 persistent homology, classes of 1-cycles generate a vector space whose dimension is equal to the number of tunnels.

The above procedure requires choosing a value of ε. A complex data set may contain multiscale empty spaces, so no single ε reveals all of them. This is particularly true for proteins, which may have essential secondary, tertiary, and quaternary structure. Persistent homology detects multiscale structure by tracking topological features across a range of ε values.

Each topological feature is present for some interval of ε values. In our example, the tunnel first appears at ε = 0.52 L. We add sufficient 2-simplices at ε = 0.99 L, so that every cycle is a boundary of one or more 2-simplices. The cycles are still present, but they are now boundaries, and so every cycle is an element of the zero class. We say that the class of cycles of interest has birth 0.52 L and death 0.99 L. We represent the tunnel by the interval [0.52, 0.99).

In general, dimension *k* persistent homology may reveal several empty spaces, each corresponding to a different interval. The dimension *k* persistent homology of such a data set is summarized by a multiset of these intervals. We call this multiset of intervals a dimension *k* barcode, and we call each interval a bar. Each bar corresponds to a class of cycles, called a persistent homology class. The dimension 1 barcode for our example is {[0.52, 0.99)}. We use the notation bc_1_(*X*) for the dimension 1 barcode of the Vietoris–Rips persistent homology of the point cloud data set *X*.

The term multiset indicates that the barcode may contain multiple copies of the same interval. For example, a perfectly symmetric figure eight would have a dimension 1 barcode consisting of two identical intervals. Biomolecules that are approximately symmetric may have pairs of similar bars corresponding to comparable topological features on either side of the molecule.

**Figure 4:**
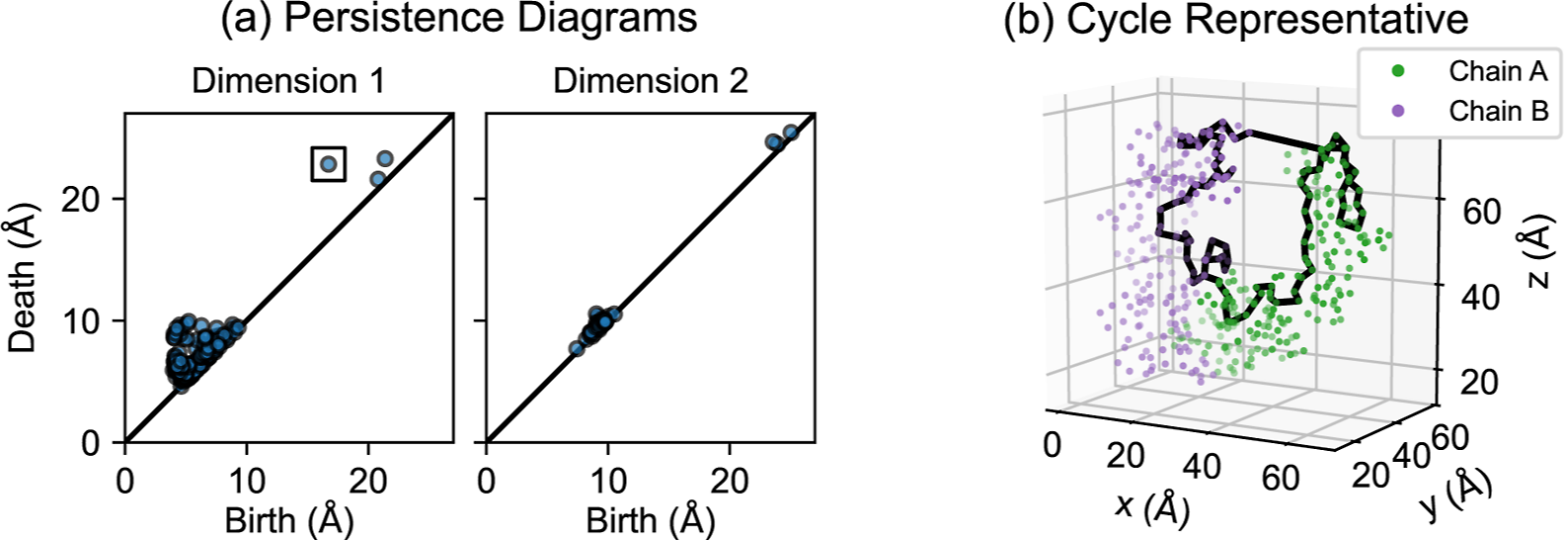
Persistence diagrams summarize the persistent homology of a data set. In this case, the data consist of spatial positions of the α carbon atoms of the NISTmAb Fc domain. (a) Points in the dimension 1 persistence diagram correspond to loops or tunnels, and points in the dimension 2 persistence diagram correspond to voids. Points farther from the diagonal of the persistence diagram indicate features which are present for larger ranges of ε. (b) This 1-cycle is a cycle representative for the class corresponding to the boxed point in the dimension 1 persistence diagram.

It can be strategic to visualize barcodes by treating the end points of each interval as the *x* and *y* coordinates of a point and producing a scatterplot. Given a dimension *k* barcode, the dimension *k* persistence diagram contains a point (*b*,*d*) for every bar [*b*,*d*) in the barcode, with the same multiplicity, as well as the diagonal, plotted as the line *y* = *x*. Dimension 1 and dimension 2 persistence diagrams for a simulated configuration of α carbon atoms in the NISTmAb Fc domain are shown in Figure 4a.

Points far from the diagonal correspond to longer intervals in the barcode, representing features which are present for wide ranges of ε. An example is the boxed point in Figure 4a, which detects the ring-like structure of the entire Fc domain. Figure 4b shows a cycle representative from this class. Local structures are present for relatively short ranges of ε, so the corresponding persistence diagram points are closer to the diagonal. In many TDA applications, short intervals in the barcode are considered noise, but for proteins they can represent true structural features.^20^

### 2.2 Betti Curves and Vectors

The first Betti number of a simplicial complex is equal to the number of loops or tunnels. As discussed in Section 2.1, this is computed by determining the dimension of the vector space generated by classes of 1-cycles. For point cloud data, we compute the first Betti number of Vietoris–Rips complexes corresponding to different values of ε.

Barcodes and persistence diagrams contain the necessary information to compute Betti numbers for any value of ε. We use the notation β_1_(ε) to denote the first Betti number at parameter value ε. We only compute first Betti numbers in the current work, so we may refer to them simply as Betti numbers.

For each nonzero class of cycles which is present in a Vietoris–Rips complex for a given value of ε, there is an interval in the barcode of the form [*b*,*d*) such that *b* ≤ ε < *d*. We can determine the Betti number by counting the number of such bars. Equivalently, we can use the persistence diagram to compute the Betti number by counting the number of off-diagonal points satisfying *x* ≤ ε and ε < *y*.

There are many ways to obtain vectors from barcodes or persistence diagrams.^31^ For a fixed point cloud *X*, and corresponding dimension 1 barcode bc_1_(*X*) or persistence diagram pd_1_(*X*), a Betti curve is a function 
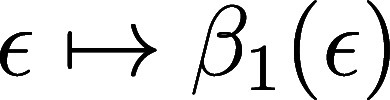
 which inputs a nonnegative real value of ε and outputs the corresponding Betti number. The Betti curve is a piecewise constant function; it is not continuous. A Betti vector is a vector
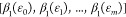
whose elements are Betti numbers evaluated at some chosen ε values ε_0_, ε_1_,..., ε_*m*_. Betti vectors consist of a discrete set of outputs of a discontinuous function; they are not stable. Informally, a small change in the input data could cause a large change in a Betti vector.

**Figure 5:**
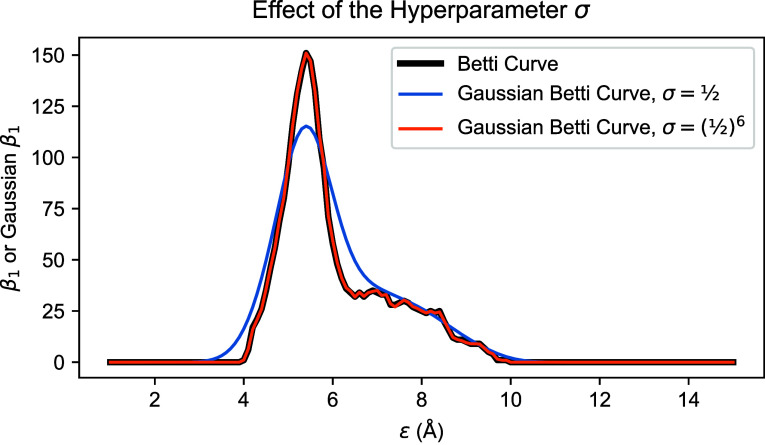
Varying degrees of smoothing can be achieved by changing the parameter σ. Smaller values of σ produce curves which are closer to the original Betti curve.

To perform smoothing, we use the strategy involved in Gaussian persistence curves;^32^ our specific implementation is described here. The general strategy, centering Gaussians at the points of a persistence diagram, is also the basis of other persistence diagram vectorizations.^33^ We address Betti vector instability by computing Gaussian Betti numbers instead of Betti numbers. Gaussian Betti numbers are values of Gaussian Betti curves, which are smooth replacements for Betti curves. To obtain a Gaussian Betti number, we center a Gaussian at each point in the off-diagonal persistence diagram. Instead of counting the number of points in a region of the *xy* plane, we integrate the sum of Gaussians over this same region. Formally, the first Gaussian Betti curve for dimension 1 persistence diagram pd_1_(*X*) and Gaussian parameter σ is given by eq 1. 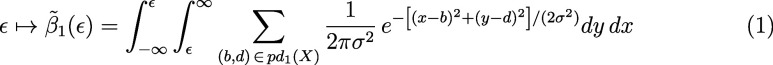
1Figure 5 shows examples of Gaussian Betti curves from the NISTmAb Fc domain computed using different values of σ. A Gaussian Betti vector is a vector
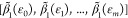
whose elements are Gaussian Betti numbers.

### 2.3 CROCKER Plots and Matrices

CROCKER plots were introduced for visualizing the topology of dynamic biological aggregations; they consist of *k*th Betti numbers β_*k*_(ε_*i*_, *t*_*j*_) evaluated at a fixed set of distances ε_*i*_ ∈ {ε_0_, ε_1_,..., ε_*m*_} and times *t*_*j*_ ∈ {*t*_0_, *t*_1_,..., *t*_*n*_}.^21^ The Betti numbers are obtained by generating a Vietoris–Rips filtration from a point cloud at each time *t*_*j*_ and computing the resulting persistent homology. Persistence is performed only with respect to the distance parameter. Persistence of topological features over time is not investigated; the Vietoris–Rips persistent homology is computed separately at each time *t*_*j*_. The computed Betti numbers are visualized in a 2-dimensional contour plot with time as the horizontal axis and the distance parameter as the vertical axis.^21^

CROCKER plots and matrices have been used for applications including the study of collective motion,^21,22,34−37^ dynamical systems bifurcations,^38^ and prediction of solar flares.^39^ CROCKER matrices may consist of Betti numbers indexed by any two parameters.^22,34^ Frequently the two parameters are time and distance,^21,22,34−37^ but other parameters may be used. Examples include a parameter associated with a dynamical system instead of time^38^ or magnetic field intensity instead of distance.^39^ In the current work, we use spatial distance and residue distance as the two parameters. The Betti numbers making up a CROCKER matrix are computed at a discrete set of values of each parameter. However, the parameters can represent either discrete or continuous quantities. In particular we note that the Vicsek model of collective motion,^40^ in which time is discrete and space is continuous, has been analyzed using CROCKER plots and matrices.^21,22^

A complication of CROCKER matrices is that, as with Betti vectors, they are not stable with respect to perturbations of the data; informally, a small change in the input data could theoretically cause a large change to the CROCKER matrix.^22,38^ A CROCKER stack is a 3-dimensional object introduced in response to CROCKER matrix instability, which performs smoothing with respect to the distance parameter.^22^ We will pursue an alternate method of smoothing, producing a 2-dimensional matrix suited for visualization.

### 2.4 Cycle Representatives

Each bar in a dimension 1 barcode or point in a dimension 1 persistence diagram corresponds to a class of 1-cycles, called a persistent homology class. An element of that class is called a cycle representative. Cycle representatives provide a way to relate topological features back to the original data set. Cycle representatives have been used to examine protein knots,^41^ protein active sites and allosteric pathways,^42^ and chromatin structure.^19^

In general there are several cycle representatives for a given bar. Different cycle representatives for the same bar may involve different points from the original data set. One way to address the nonuniqueness of cycle representatives is to obtain an optimal cycle representative rather than an arbitrary cycle representative.

Identifying optimal cycle representatives, for example using linear programming approaches,^43^ is a substantial area of research. There may be multiple reasonable definitions of what constitutes an optimal cycle. For sequential data sets, selection of cycle representatives can incorporate information about the vertex order; this approach has applications to chromatin structure^19^ and animal trajectories.^44^ The choice of what to consider as optimal may alter the cycle representative which is identified. For example, in Figure 3b, cycle A encloses a smaller area, but cycle B is shorter in length. Implementation details such as the choice of constraints or linear solver may also affect the optimization.^43^ One needs to exercise caution when interpreting cycle representatives, whether or not optimization is used. We use cycle representatives for visualization purposes only.

## 3 Methods

**Figure 6:**
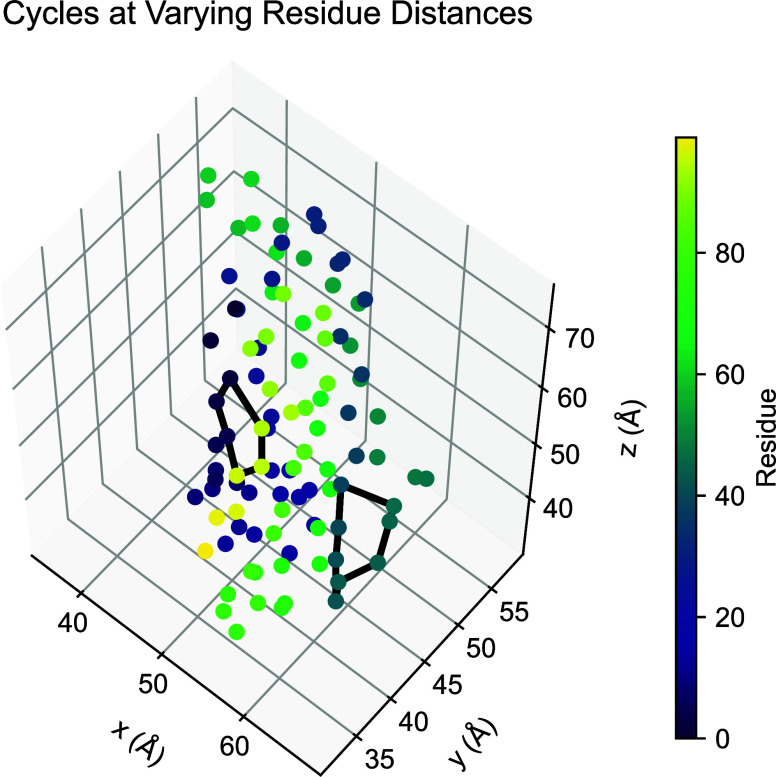
α carbon atoms corresponding to the first 100 residues of a simulated conformation of the NISTmAb Fc domain are shown, with the color of the points indicating the relative sequential position of each residue. Two cycle representatives are shown, one (on the right) containing α carbons from residues in a single segment of an amino acid chain, and the other (on the left) corresponding to residues from two sequentially distant segments of the amino acid chain. The left cycle lies at the site of an immunoglobulin fold between two neighboring β sheets.

Our approach is motivated by the variation in sequence composition of different cycles in the NISTmAb Fc domain. Some cycles, including the one shown in Figure 4b, contain vertices corresponding to residues from both amino acid chains. Other cycles contain vertices corresponding to residues from a single amino acid chain. Figure 6 shows two such cycles.

Residues belonging to the same amino acid chain have residue numbers indicating their relative positions in the sequence of amino acids making up the chain. The residue distance between two α carbon atoms in the same chain is the absolute difference between the corresponding residue numbers. For the left cycle in Figure 6, the largest residue distance between residues corresponding to adjacent vertices in the cycle is 92. This cycle lies at the site of an immunoglobulin fold between two neighboring β sheets. For the right cycle, the largest distance is 7. The left cycle forms where different parts of the chain have folded to become physically close to each other, while the right cycle only involves a limited portion of the chain.

In persistent homology, the objects of interest are classes of cycles. We can consider the sequence composition of the cycles belonging to a given persistent homology class. For some classes, every cycle representative contains vertices from both amino acid chains. An example would be the persistent homology class shown in Figure 4. These persistent homology classes are related to the quaternary structure of the protein.

Other persistent homology classes have at least one cycle representative whose vertices correspond to residues from a single amino acid chain. For some persistent homology classes of this type, all cycles restricted to a single chain require some connections between vertices corresponding to large residue distances. For other classes of this type, the persistent homology class contains some cycle representatives for which all residue distances corresponding to adjacent vertices are small.

Our goal is to separate topological features by sequence composition without the need for cycle optimization or thorough characterization of the cycles belonging to each persistent homology class. We do so by tracking changes in Gaussian Betti curves. These changes are recorded in the GCCD matrix. In this section we outline our methods, including data set generation through molecular dynamics simulations, GCCD matrix construction, and subsequent analysis.

### 3.1 Data Sets

The data consist of four simulated glycosylated trajectories and four simulated aglycosylated trajectories of the NISTmAb Fc domain. Bergonzo et al. describe the molecular dynamics force fields and parameters.^10^ The glycosylated starting structures are based on the structure from PDB code 5VGP(45,46) and the aglycosylated starting structures are based on the structure from PDB code 7RHO.^10,47^ The trajectories are numbered 0, 1, 2, and 3. The trajectory numbering is arbitrary. For example, glycosylated trajectory 0 and aglycosylated trajectory 0 have no connection to each other.

The glycosylated and aglycosylated simulations differ due to the presence or absence of glycans and the types of starting structures used to initialize each trajectory. Within a simulation type (glycosylated or aglycosylated), trajectories differ in their starting structure. Each trajectory consists of 1000 frames (downsampled from 10,000 frames); we omit the initial frames of each trajectory from our analysis, allowing the molecule to move away from the selected starting conformation. We report results for starting frames of 200, 350, and 500. Consecutive frames considered in this analysis correspond to 1 ns of elapsed simulation time. All atoms are used to generate the molecular dynamics simulations, but we only use the α carbon atoms to perform TDA. Coarse-grain models of the NISTmAb are also available,^48^ and may be of interest for exploring larger-scale topological structures in future work. The 3-dimensional coordinates of α carbon atoms are given in units of angstroms (Å), and the Euclidean distances between the spatial positions of atoms are also given in angstroms (Å).

In addition to our main focus of molecular dynamics simulations, we also generate GCCD matrix summaries for the experimentally based conformations 5VGP(45,46) assembly 1 and 7RHO(10,47) assemblies 1, 2, and 3. For consistency, we analyze the same residues across all data sets. The first residue included in the analysis is Pro241, and the last residue is Leu446. These are the same residues analyzed for the molecular dynamics simulations.

### 3.2 Vietoris–Rips Filtrations

For simulated NISTmAb conformations, the data points are equipped with both a spatial distance and a residue distance. The spatial distance between points is the Euclidean distance between the 3-dimensional coordinates of the α carbon atoms. We define the residue distance between points in the same amino acid chain to be the absolute difference between the positions of the residues in the amino acid sequence. In the simulated conformations we analyze, each chain contains 206 residues, so the maximum possible residue distance is 205.

We produce a sequence of Vietoris–Rips filtrations, one for each residue distance from 1 to 205. An additional Vietoris–Rips filtration allows connections between any vertices, even those corresponding to residues from different chains. This final filtration is a classical Vietoris–Rips filtration which considers only the 3-dimensional coordinates of the α carbon atoms. For bookkeeping purposes, we can define the residue distance between points corresponding to α carbon atoms from different amino acid chains to be 206. Then we obtain a Vietoris–Rips filtration for each residue distance from 1 to 206, and we do not need a separate naming convention for the final, classical Vietoris–Rips filtration. Defining a residue distance between α carbons in different chains is convenient, but not necessary; it would be equally valid to characterize connections based on “residue distance or chain membership,” where we allow connections between different chains only after we allow connections between α carbon atoms with arbitrarily high within-chain residue distances.

A Vietoris–Rips filtration depends on the pairwise dissimilarities between points, so to generate each filtration it suffices to define the dissimilarity between any two α carbon atoms. Suppose two α carbon atoms *a*_1_ and *a*_2_ have coordinates (*x*_1_, *y*_1_, *z*_1_) and (*x*_2_, *y*_2_, *z*_2_) and correspond to residues from chains *c*_1_ and *c*_2_ with residue numbers *r*_1_ and *r*_2_. For a fixed residue distance 1 ≤ ρ ≤ 205, we define the dissimilarity between the α carbons to be
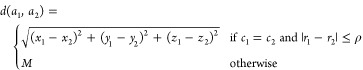
where *M* is some large value. Specifically, we set *M* to be larger than the maximum pairwise dissimilarity for which persistent homology is computed. This effectively forbids connections between certain vertices.

### 3.3 GCCD Matrices

**Figure 7:**
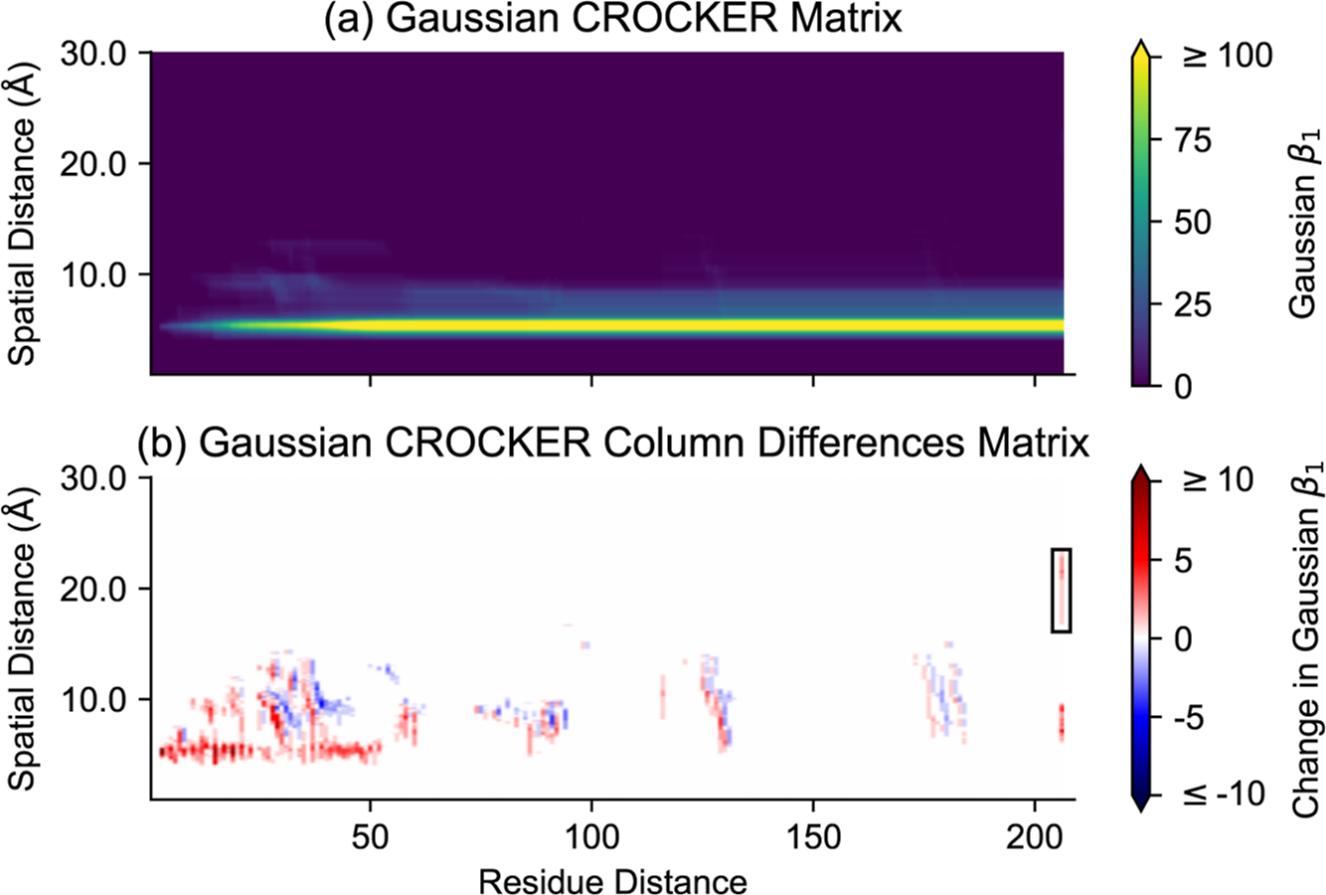
One approach to producing vectorized topological summaries is to construct a vector or matrix whose elements are Betti numbers. The first Betti number is equal to the number of loops or tunnels in a simplicial complex. (a) A CROCKER matrix shows Betti numbers which depend on two parameters, in this case spatial distance and residue distance. Each element of such a CROCKER matrix is the number of loops or tunnels in a simplicial complex formed by connecting sets of α carbon atoms which are pairwise sufficiently close with respect to both spatial and residue distances. A Gaussian CROCKER matrix incorporates smoothing with respect to the spatial distance. (b) We obtain a Gaussian CROCKER Column Differences (GCCD) matrix from a Gaussian CROCKER matrix by taking differences between successive columns. A GCCD matrix makes many topological features more apparent. For example, the boxed region at the right of the GCCD matrix contains the signal generated by the class of cycles discussed in Figure 4.

We incorporate both geometric and sequence information in a topological summary which we call a Gaussian CROCKER column differences (GCCD) matrix. This matrix is obtained from a Gaussian CROCKER matrix. Examples of a Gaussian CROCKER matrix and GCCD matrix are shown in Figure 7a,b.

Each column of a Gaussian CROCKER matrix is a Gaussian Betti vector, which is a smooth replacement for a Betti vector. This process requires a choice of Gaussian, controlled by the hyperparameter σ. Smaller values of σ produce Gaussian Betti vectors which are closer to the original Betti vector. See Figure 5 for example results for different choices of σ.

For Gaussian CROCKER matrices, the Gaussian Betti numbers are evaluated at a sequence of values of ε. We use a regular grid of ε values
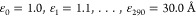
such that the smallest ε value in the grid is chosen to be smaller than the minimum cycle birth seen in a typical barcode, and the largest ε value in the grid is chosen to be larger than the maximum cycle death seen in a typical barcode.

We accomplish this by randomly selecting 1000 frames (out of the four glycosylated and four aglycosylated trajectories) and computing the dimension 1 persistent homology for each frame. The smallest such birth is 3.87 Å, while the largest death is 27.5 Å. Rounding the smallest birth down to 3 Å and the largest death up to 28 Å, and extending these values by a buffer of 2 Å (four times the largest value of σ used for smoothing), yields a range of 1–30 Å. The total time needed to perform this preliminary step was about 15 min on a laptop. For the experimental data, the smallest birth is 3.89 Å and the largest death is 26.6 Å. Based on these values, the same range of ε used for analyzing the molecular dynamics simulations is appropriate for generating GCCD matrices from the experimental data.

When constructing GCCD matrices, we compute persistent homology up to the largest value of ε, plus some small value to avoid numerical issues (we add 0.001 Å). Any class of cycles still present when we stop computing persistent homology is assigned a death of 30.001.

A Gaussian CROCKER matrix is a smooth replacement for a CROCKER matrix, addressing CROCKER matrix instability. For low values of σ, the Gaussian CROCKER matrix is similar to the original CROCKER matrix. By treating σ as a tunable hyperparameter, we may be able to improve the final classification accuracy for distinguishing glycosylated versus aglycosylated conformations. Note that we only perform smoothing with respect to the spatial distance parameter. We do not perform smoothing with respect to the residue distance parameter, but residue distances are discrete. Specifically, they are integer valued. We include a matrix column for every positive integer valued residue distance until every possible connection is allowed. We do not check for persistence of topological features with respect to changing residue distances, but rather we compute a separate Vietoris–Rips filtration for each residue distance. We do utilize persistent homology with respect to spatial distance, as the birth and death values of each bar are used to obtain the Gaussian Betti numbers.

Different classes of cycles emerge at different residue distances. For example, Figure 6 suggests that the class of cycles whose cycle representative is shown on the right (with teal vertices) emerges at a smaller residue distance than the class of cycles whose cycle representative is shown on the left (with yellow and purple vertices). If this is the case, the teal class of cycles would affect more columns of the Gaussian CROCKER matrix than the yellow/purple class. One way to mitigate this effect is to subtract successive Gaussian CROCKER matrix columns. This produces the Gaussian CROCKER column differences (GCCD) matrix.

The GCCD matrix records the change in Gaussian Betti numbers with respect to residue distances. We can think of this matrix as a representation of the accumulation of classes of cycles as we progressively allow connections between residues that are farther and farther away from each other in the amino acid sequence.

This also has benefits in terms of visualization. For example, we can see from the GCCD matrix in Figure 7b that many classes of cycles surrounding small empty spaces accumulate at a range of residue distances, up to a residue distance of about 50. We also reveal certain topological features that are visible in the persistence diagram but not visually apparent in the Gaussian CROCKER matrix due to high Gaussian Betti numbers in some parts of the matrix. See the boxed region in Figure 7b for an example.

### 3.4 Vector Summaries of Spatial Topology

A defining feature of the GCCD matrix is that it incorporates both spatial and sequential information. We will compare the performance of classification using GCCD matrices to the performance of classification using vectorized topological summaries which only incorporate spatial information. The first topological summary we use for comparison is the Gaussian Betti curve (for dimension 1 persistent homology). We can obtain this summary from the GCCD matrix (for dimension 1 persistent homology) by adding the columns. We also check a combined topological summary which includes Gaussian Betti curves for persistent homology in dimensions 0, 1, and 2. Curves for different dimensions may have different scales, so we normalize each curve. One recommended normalization method, which we adopt, is to divide by the number of points in the persistence diagram for the relevant dimension.^49^ Normalized Gaussian Betti curves cannot be obtained from a GCCD matrix, because the GCCD matrix does not record the number of persistence diagram points. We compute the normalized Gaussian Betti curves for dimensions 0, 1, and 2 separately, and concatenate them.

### 3.5 Multidimensional Scaling (MDS)

GCCD matrices and the other vectorized (vector or matrix) topological summaries we consider are high-dimensional objects, which makes it difficult to visualize collections of GCCD matrices for a trajectory or set of trajectories. For *k*-nearest neighbors classification, we use the pairwise distances between GCCD matrices. We can use multidimensional scaling (MDS),^50^ which finds an optimal set of points in 2-dimensional space whose pairwise distances approximate the actual pairwise distances between GCCD matrices. This allows us to have some intuition regarding how the classification will perform.

### 3.6 Classification

We use *k*-nearest neighbors classification to predict whether test conformations from molecular dynamics simulations are glycosylated or aglycosylated. For each frame of a molecular dynamics simulation, we compute a vectorized topological summary. We can quickly compute pairwise Euclidean distances between these summaries. We predict whether a frame from a test trajectory is glycosylated or aglycosylated by computing its topological summary, finding the *k* closest topological summaries from the training data, and taking a majority vote. The best value of *k* is chosen through a hyperparameter grid search. Potential values of *k* are all chosen to be odd, to avoid potential ties. For the experimental data, the number of conformations is not large enough to permit *k*-nearest neighbors classification analysis, but we use principal component analysis (PCA) to explore possible separability of experimental glycosylated versus aglycosylated conformations.

### 3.7 Hyperparameter Grid Search

For the molecular dynamics data, we use a grid search to find the best combination of hyperparameter values. Computing a Gaussian Betti vector requires choosing a value of σ. We test hyperparameter values of , and . The number of experimental conformations we analyze is not high enough to permit parameter tuning, so we use a moderate value of  for GCCD matrices generated from experimental structures.

When performing *k*-nearest neighbors classification we select a value of *k*. We test values of *k* = 15, 25, 35, and 45. Note that we use the term hyperparameter for σ and *k* while we follow the convention of using the term parameter for the values that determine each simplicial complex and its (Gaussian) Betti numbers. In the current work, these parameters are residue distance (with the largest residue distance indicating membership in different amino acid chains) and spatial distance.

For each train/test split, we select the best hyperparameters for the training data. Each training data set consists of three glycosylated trajectories and three aglycosylated trajectories. We perform cross-validation within each training data set to select hyperparameters. We form nine train/validation splits within each training data set, each time leaving out one glycosylated trajectory and one aglycosylated trajectory for validation. We select the pair of hyperparameters which yields the highest mean validation accuracy.

### 3.8 Test Accuracies

For each type of topological summary, we perform 16 train/test splits. In each data set split, we select a single glycosylated trajectory and a single aglycosylated trajectory to act as the testing data. The remaining trajectories serve as the training data. We use the hyperparameters identified using each training data set as described in Section 3.7. We determine the mean test accuracy for each data set split.

### 3.9 Software

We provide GCCD implementations in two programming languages. The first is written in the Julia programming language.^51^ We use the packages DataFrames^52^ for data management, Eirene^53^ for topological data analysis, and distributions^54,55^ for integrating Gaussians to obtain Gaussian Betti vectors. The second implementation is written in Python using the packages pandas^56,57^ for data management, Open Applied Topology (OAT)^58^ for TDA, and NumPy^59^ and SciPy^60^ for computations.

For every data set (glycosylated and aglycosylated), trajectory (0, 1, 2, and 3), and frame (200 through 999), and for every hyperparameter value σ included in the grid search, we compute a GCCD matrix using both the Julia implementation and the Python implementation. We round to ten decimal places when saving the results.

We use cycle representatives for visualization. All cycle representatives are obtained in Julia,^51^ using the package Eirene.^53^ For Python users, Open Applied Topology^58^ can also return cycle representatives. Since cycle representatives are only used for visualization, researchers interested in utilizing GCCD matrices do not need to limit themselves to TDA software options which have the ability to compute these.

After performing topological data analysis, we use Python with NumPy^59^ for subsequent data analysis and plotting. We use pandas^56,57^ for data management. We use Matplotlib^61^ for producing plots. For some visualizations we use convex hulls, which are computed in SciPy^60^ based on Qhull.^62^ We perform MDS and *k*-nearest neighbors using scikit-learn.^63^

To obtain GCCD matrices, it is only necessary to be able to obtain barcodes for Vietoris–Rips filtrations using a dissimilarity matrix as input. This means there are several software options for researchers interested in utilizing GCCD matrices. The Julia and Python implementations we provide are not the only possibilities. Otter et al. investigate some TDA software options, including computing Vietoris–Rips persistent homology from a distance matrix.^64^

## 4 Results

**Figure 8:**
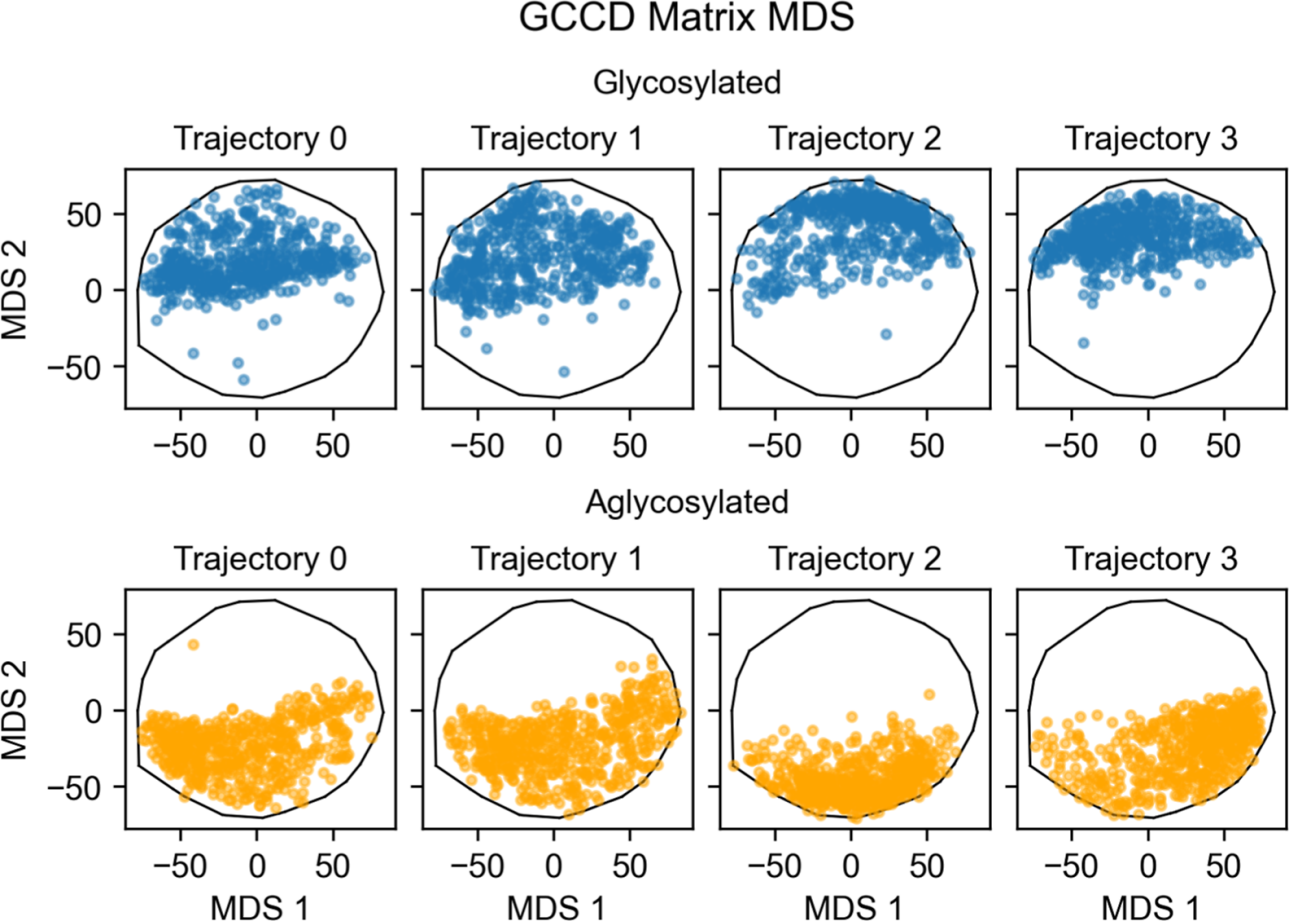
Multidimensional scaling of GCCD matrices from each trajectory, starting at frame 500. Each point represents a single GCCD matrix, computed using . The black outline is the border of the convex hull of all points.

**Figure 9:**
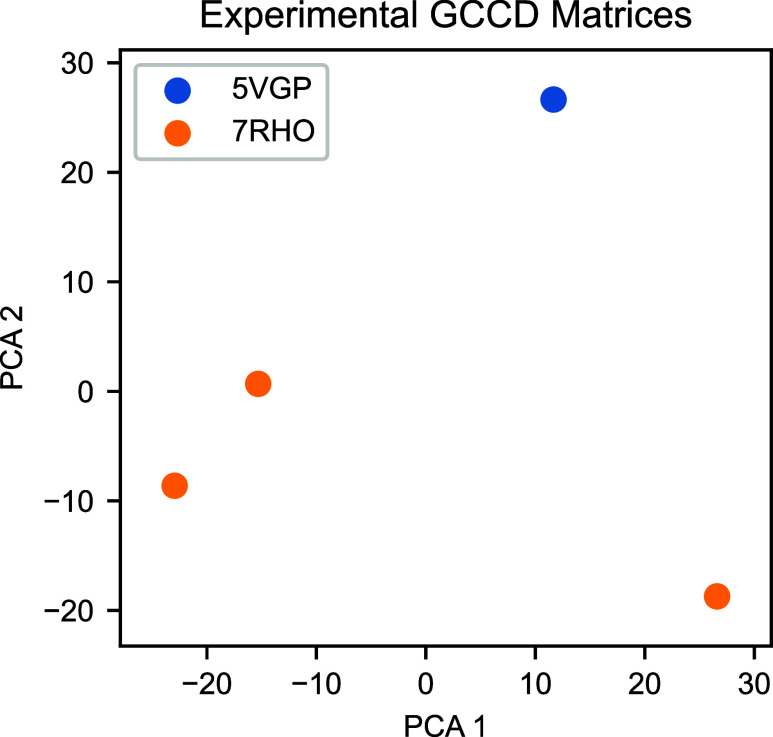
PCA of GCCD matrices for experimental conformations, computed using . The first two PCA components have explained variance ratios of 0.469 and 0.332, respectively.

Element-wise comparisons of every pair of matrices from the Julia and Python implementations found that every absolute difference between corresponding matrix elements was less than 1.0001 × 10^–10^. Hyperparameter tuning results are shown in the Supporting Information. The classification performance appears to be relatively robust to the choice of hyperparameters. Figure 8 shows the MDS results. Mean test accuracies are shown in Table 1. Each value is an average across all train/test splits. Test accuracy results for individual train/test splits are reported in the Supporting Information. Figure 9 shows the GCCD matrix PCA results for experimental conformations.

**Table 1:** Classification Test Accuracies

	starting frame		
topological summary	200	350	500
Gaussian Betti curve (1D)	0.715	0.717	0.704
Gaussian Betti curves (0D, 1D, 2D)	0.685	0.695	0.665
GCCD matrix (1D)	0.931	0.946	0.966

## 5 Discussion

As shown in Table 1, GCCD matrices yield better *k*-nearest neighbors classification accuracies than Gaussian Betti curves. This remains true even when the Gaussian Betti curves include results from dimension 0 and dimension 2 persistent homology, information which is not incorporated in the GCCD matrix. The mean test accuracies are slightly lower when incorporating dimension 0 and dimension 2 persistent homology, perhaps because there is more noise in one of these dimensions.

GCCD matrix classification accuracy appears to improve somewhat as we restrict to later portions of the trajectory. This is encouraging, because it suggests the results are not dependent on the choice of starting conformations. This potential trend could be explored more in future work through the use of additional and longer trajectories.

Figure 9 shows the GCCD matrix PCA results for experimental conformations. The glycosylated and aglycosylated conformations are separated along the second PCA coordinate. The differences between conformations may reflect the difference in space groups in deposited crystallized structures as well as changes due to glycosylation. Given the dynamic nature of IgG1 Fc domains, we would need larger numbers of structures to confidently characterize the efficacy of GCCD matrix classification of experimental data.

**Figure 10:**
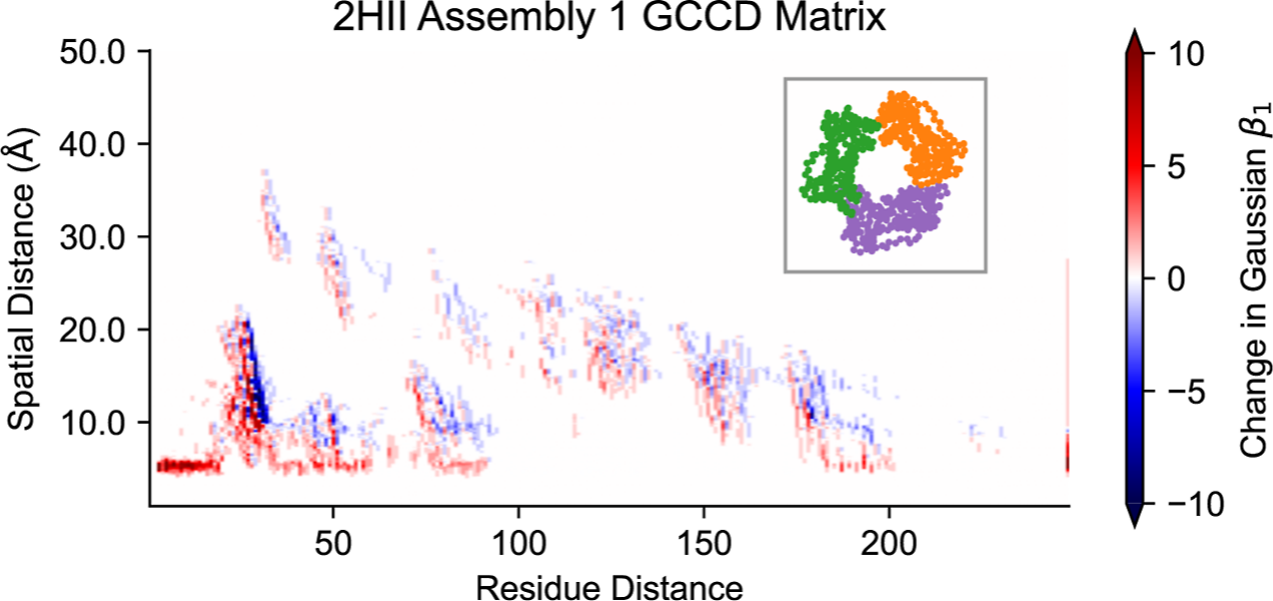
GCCD matrices can be generated from many structures beyond Fc domains. This GCCD matrix summarizes the spatial and sequential topology of the PCNA clamp protein whose α carbon positions are shown in the inset, with chains A, B, and C plotted in purple, orange, and green, respectively. The matrix was computed from the first assembly in the 2HII PDB file.

This work focuses on molecular dynamics simulations of IgG1 Fc domains, but the general framework can be applied to other data formats and protein systems. As an example, we generate a GCCD matrix from PDB structure 2HII^65,66^ of a PCNA clamp protein. After formatting the data (code to accomplish this included in the accompanying repository), we use the same function which was used for generating the Fc domain GCCD matrices. The result is shown in Figure 10. The positive values in the last column signify the emergence of topological features which require more than one chain, such as the overall loop structure of the protein which involves all three chains. For proteins with three or more amino acid chains, more comparisons are possible, such as comparing features requiring at most two chains to features requiring all three chains. We leave detailed analysis of other systems for future work.

Our analysis is aided by the fact that we are comparing simulations generated from the same protein sequence (but differing due to the presence or absence of glycans). In this context, residue distances from different simulations are directly comparable. It is possible to use these techniques in more general contexts. Sequence alignment may facilitate the use of GCCD matrices for the comparison of proteins which do not have identical sequences. GCCD matrices can still be constructed without first performing sequence alignment, but similar topological features may appear in different columns of the matrices. This could be addressed through the use of an appropriate notion of distances between matrices.

In future work, we could pursue feature selection with respect to both spatial distance and residue distance. One option would be to optimally select elements or regions of the GCCD matrix. Another would be to alter the matrix construction to emphasize certain classes of cycles. The smoothing we perform can be combined with weighting. Formally, we can replace the Gaussian Betti curve computation in eqs 1 with 2. 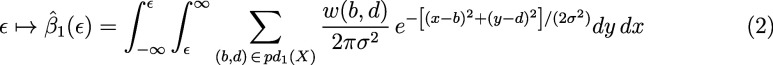
2Equation 1 can be obtained from eq 2 by setting *w*(*b*,*d*) = 1. By altering the weight function *w*(*b*,*d*) assigned to each bar [*b*,*d*), we can prioritize cycle classes based on their births, deaths, or lifetimes (lifetime = death – birth).

**Figure 11:**
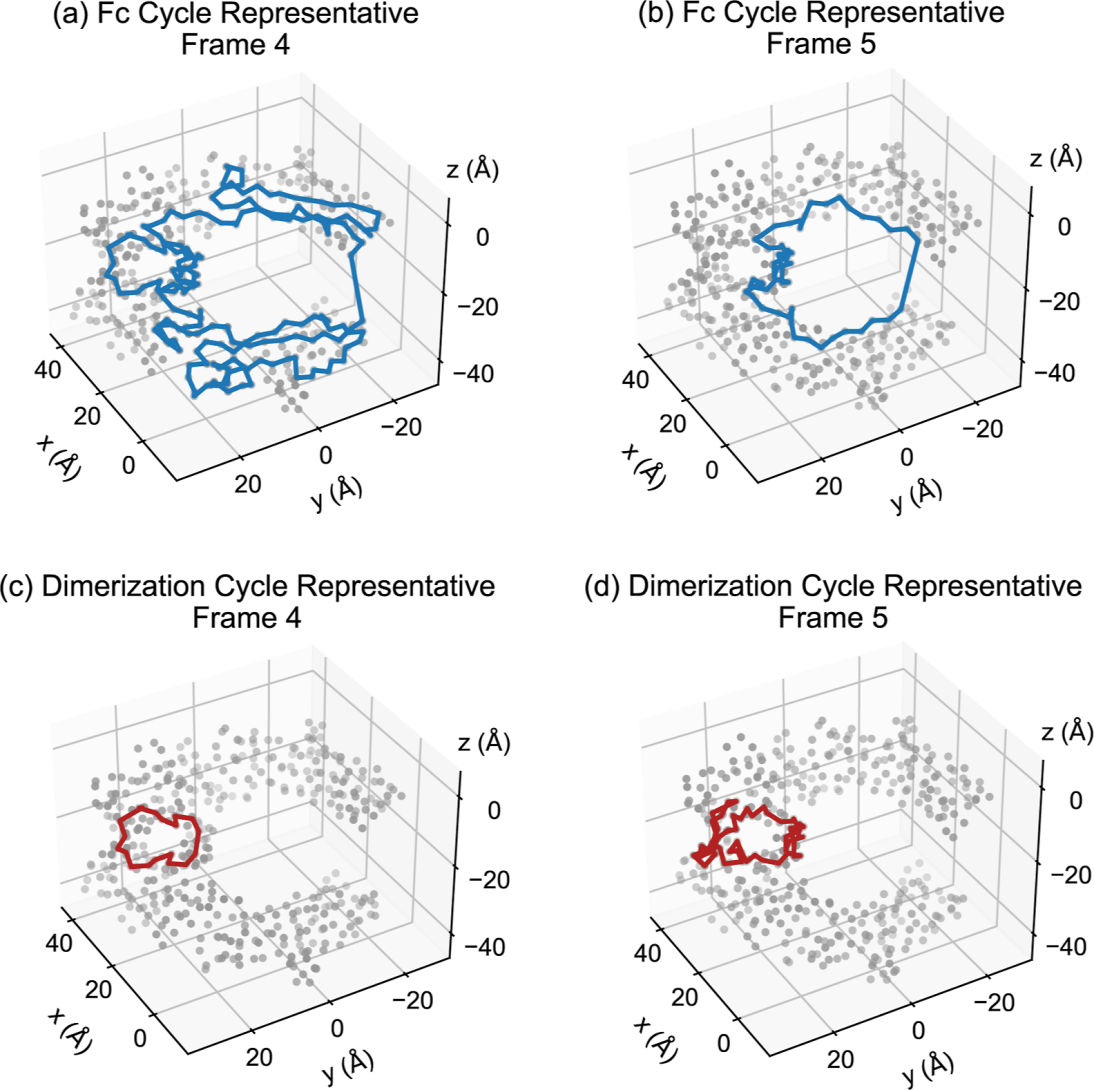
We examine two simulated glycosylated conformations of the NISTmAb Fc domain from trajectory 1, separated by one ns of simulation time. The α carbon atoms are plotted in gray. For each frame, a cycle representative of the most persistent class of 1-cycles, corresponding to the tunnel structure of the entire Fc domain, is plotted in blue. A cycle representative for the dimerization region is plotted in red. These are the default cycle representatives returned by Eirene.^53^ (a) The Fc cycle representative returned for frame 4 traverses many local protein structures, including surrounding the dimerization region. (b) The Fc cycle representative returned for frame 5 takes a path closer to the interior of the domain. (c) The dimerization cycle representative from frame 4 is relatively simple, containing only 19 edges. (d) The dimerization cycle representative from frame 5 contains 52 edges.

Given the success of GCCD matrices for classification of glycosylated versus aglycosylated conformations, one may be interested in other types of topological descriptors which utilize both spatial and sequential information. An existing object which can incorporate sequence information is the cycle representative. Unfortunately, cycle representatives can be complicated to work with. A cycle representative is a single element from a class of cycles, and there is not a unique choice of cycle representative. In Figure 11, we show examples of cycle representatives for comparable features in adjacent frames of a molecular dynamics simulation. These cycle representatives vary greatly in terms of, for example, the number of simplices contained in each cycle. It can be difficult to use cycle representatives as inputs for analysis tasks such as classification when they differ not just due to changes in the underlying topology, but also due to changes in the choice of a representative from the cycles in a class.

For the GCCD matrices generated from Fc domains, we consider 412 α carbon atoms distributed across 2 chains, and construct the matrix up to a maximum spatial distance 30 Å. For the GCCD matrices generated from a clamp protein, we consider 718 α carbon atoms distributed across 3 chains, and construct the matrix up to a maximum spatial distance 50 Å. Run time statistics for the Fc domains are shown in Table 2, and run time statistics for the clamp protein are shown in Table 3. For the Fc domains, we timed computations for 20 randomly selected frames. For the clamp protein, we timed the conformation shown in Figure 10. We performed 7 runs for each structure on a laptop. Individual run times are reported in the Supporting Information.

**Table 2:** Time in Seconds to Read Formatted Data from One Frame of an Fc Domain Molecular Dynamics Simulation and Produce a GCCD Matrix for Each Value of σ Included in the Grid Search, for a Random Selection of Frames

	Fc domain run time (s)		
implementation	minimum	median	maximum
Julia	18.3	20.3	22.3
Python	38.7	39.7	41.2

**Table 3:** Time in Seconds to Read Formatted Data for a PCNA Clamp Protein and Produce a GCCD Matrix for Each Value of σ Included in the Fc Domain Classification Grid Search

	2HII run time (s)		
implementation	minimum	median	maximum
Julia	92.6	94.4	98.0
Python	166.9	169.5	173.2

The clamp protein is a larger data set, leading to longer run times. The Python implementation had longer run times than the Julia implementation. The run times we report are for our implementations of GCCD matrix construction, and do not reflect the potential performance of the programming languages or utilized packages for other tasks. Other factors, such as the persistent homology dimension and available computing power, can also affect run times. A full characterization of possible GCCD computations is beyond the scope of this work, but we provide run times for the matrices we constructed as a guide for other potential users. Our implementations compute the columns of the Gaussian CROCKER matrix independently. In future implementations, we could explore constructing these columns in parallel to decrease run times.

In addition to approaches using TDA, clustering techniques can also incorporate both spatial and sequential distances; see, for example, an application to chromosome structure.^67^ Techniques for tracking topological features across scales can be used for hierarchical clustering. Our approach can detect the number of clusters at different spatial and residue scales through the use of dimension 0 persistent homology (we focus on dimension 1 persistent homology in this work, rather than dimension 0 persistent homology which produces hierarchical clusters).

Dimension 1 persistent homology detects loops or tunnels. Topological analysis of selected frames suggests that voids are less common than tunnels and that when voids are present, they persist for smaller intervals of the spatial distance parameter. See, for example, Figure 4. Considering a single persistent homology dimension simplifies the analysis, but in some contexts it may be best to consider every available dimension. For a biomolecule, one could use the dimension 0, 1, and 2 persistent homology to obtain GCCD matrices summarizing the component, tunnel, and void structure, respectively. These three matrices can be treated like three channels of a color image, enabling subsequent analysis with machine learning techniques tailored for image processing.

## 6 Conclusions

Topological data analysis enables quantification of features which are not easily identified by classical analysis methods. A common starting point for topological analysis is a finite collection of data points equipped with pairwise distances or dissimilarities between points. In many biological applications, such as simulations of biomolecules, sequence information is also available. This may consist of residue numbering and/or association with a particular subchain. Leveraging this additional information can improve topological descriptions of biological systems. By using residue distances in GCCD matrix construction, we incorporate sequence information which is not utilized in typical persistent homology analysis of point clouds in Euclidean space.

GCCD matrices inherently capture structure across both spatial and residue distance scales. We do not require user selection of a fixed spatial or residue distance threshold. The data required to generate a GCCD matrix consist of the pairwise spatial and residue distances between data points (in our case, α carbon atoms). Since the analysis is based on pairwise distances, we do not select a set of reference axes or directions.

The essential step in computing a GCCD matrix is obtaining the barcodes of a collection of Vietoris–Rips filtrations. Vietoris–Rips persistent homology is one of the most common TDA techniques, and is implemented in several TDA software options. This has many advantages, for example allowing researchers to continue using one of their preferred programming languages. We provide implementations in Julia and Python.

Once we computed the GCCD matrices, we obtained good classification of the available simulated glycosylated versus aglycosylated conformations. We only needed to tune two hyperparameters, namely σ which determines the degree of smoothing and *k* which is the number of neighbors used for classification. During hyperparameter tuning, the best and worst mean validation accuracies across hyperparameter choices for a given validation data set did not differ greatly, suggesting that classification using GCCD matrices is relatively robust to the choice of hyperparameters. Furthermore, classification using GCCD matrices produced better test accuracies than classification using standard topological vectorizations which only incorporate spatial distance information. We are optimistic that GCCD matrices are a method of spatial and sequential feature extraction which can be efficiently utilized by other researchers.

## Data Availability

The data sets are described in Section 3.1. Formatted data and code are available at https://github.com/usnistgov/NISTmAb-TDA.

## Supporting Information Available

Hyperparameter selection results, test accuracies for individual train/test splits, and individual run times (PDF)

## Notes

Certain software are identified in this paper in order to specify the data analysis procedure adequately. Such identification is not intended to imply recommendation or endorsement of any product or service by NIST, nor is it intended to imply that the software identified are necessarily the best available for the purpose.
