# Supplementary material for: Spatial and Sequential Topological Analysis of Molecular Dynamics Simulations of IgG1 Fc Domains
Source: J Chem Theory Comput. 2025 Apr 22;21(9):4884–97. doi: 10.1021/acs.jctc.5c00161 (PMC12079798; doi:10.1021/acs.jctc.5c00161)
Supplement: Supplementary file 1 — ct5c00161_si_001.pdf [file ct5c00161_si_001.pdf]

Supporting Information

Spatial and Sequential Topological Analysis of Molecular Dynamics Simulations of IgG1 Fc Domains

Melinda Kleczynski,[†] Christina Bergonzo,[*,†,‡] and Anthony Kearsley[*,†]

†*National Institute of Standards and Technology, Gaithersburg, MD 20899, USA*

‡*Institute for Bioscience and Biotechnology Research, Rockville, MD 20850, USA*

E-mail: christina.bergonzo@nist.gov; anthony.kearsley@nist.gov

List of Tables

S1 Hyperparameter selection results for Gaussian Betti curves, starting at frame 200, labeled by training trajectories. S3

S2 Hyperparameter selection results for Gaussian Betti curves, starting at frame 350, labeled by training trajectories. S4

S3 Hyperparameter selection results for Gaussian Betti curves, starting at frame 500, labeled by training trajectories. S5

S4 Hyperparameter selection results for concatenated normalized 0D, 1D, and 2D Gaussian Betti curves, starting at frame 200, labeled by training trajectories. S6

S5 Hyperparameter selection results for concatenated normalized 0D, 1D, and 2D Gaussian Betti curves, starting at frame 350, labeled by training trajectories. S7

S6 Hyperparameter selection results for concatenated normalized 0D, 1D, and 2D Gaussian Betti curves, starting at frame 500, labeled by training trajectories. S8
S7 Hyperparameter selection results for GCCD matrices, starting at frame 200, labeled by training trajectories. S9
S8 Hyperparameter selection results for GCCD matrices, starting at frame 350, labeled by training trajectories. S10
S9 Hyperparameter selection results for GCCD matrices, starting at frame 500, labeled by training trajectories. S11
S10 Test accuracies for Gaussian Betti curves, starting at frame 200. . . . . S12
S11 Test accuracies for Gaussian Betti curves, starting at frame 350. . . . . S13
S12 Test accuracies for Gaussian Betti curves, starting at frame 500. . . . . S14
S13 Test accuracies for concatenated normalized 0D, 1D, and 2D Gaussian Betti curves, starting at frame 200. . . . . S15
S14 Test accuracies for concatenated normalized 0D, 1D, and 2D Gaussian Betti curves, starting at frame 350. . . . . S16
S15 Test accuracies for concatenated normalized 0D, 1D, and 2D Gaussian Betti curves, starting at frame 500. . . . . S17
S16 Test accuracies for GCCD matrices, starting at frame 200. . . . . S18
S17 Test accuracies for GCCD matrices, starting at frame 350. . . . . S19
S18 Test accuracies for GCCD matrices, starting at frame 500. . . . . S20
S19 Fc Domain GCCD matrix run times in seconds (s) in Julia. S21
S20 Fc Domain GCCD matrix run times in seconds (s) in Python. S22
S21 2HII GCCD matrix run times in seconds (s) in Julia. S23
S22 2HII GCCD matrix run times in seconds (s) in Python. S23

Topological summaries are generated from dimension 1 persistent homology unless otherwise noted.

Table S1: Hyperparameter selection results for Gaussian Betti curves, starting at frame 200, labeled by training trajectories.

Glycosylated Trajectories	Aglycosylated Trajectories	Best Hyperparameters	Best Mean Accuracy	Worst Mean Accuracy
1, 2, 3	1, 2, 3	$\sigma = \left(\frac{1}{2}\right)^4, k = 45$	0.695	0.664
1, 2, 3	0, 2, 3	$\sigma = \left(\frac{1}{2}\right)^5, k = 35$	0.735	0.697
1, 2, 3	0, 1, 3	$\sigma = \left(\frac{1}{2}\right)^4, k = 45$	0.644	0.620
1, 2, 3	0, 1, 2	$\sigma = \left(\frac{1}{2}\right)^5, k = 35$	0.677	0.650
0, 2, 3	1, 2, 3	$\sigma = \left(\frac{1}{2}\right)^3, k = 45$	0.742	0.720
0, 2, 3	0, 2, 3	$\sigma = \left(\frac{1}{2}\right)^3, k = 45$	0.775	0.746
0, 2, 3	0, 1, 3	$\sigma = \left(\frac{1}{2}\right)^2, k = 45$	0.710	0.687
0, 2, 3	0, 1, 2	$\sigma = \left(\frac{1}{2}\right)^3, k = 35$	0.734	0.715
0, 1, 3	1, 2, 3	$\sigma = \left(\frac{1}{2}\right)^3, k = 45$	0.707	0.675
0, 1, 3	0, 2, 3	$\sigma = \left(\frac{1}{2}\right)^3, k = 45$	0.749	0.705
0, 1, 3	0, 1, 3	$\sigma = \left(\frac{1}{2}\right)^3, k = 45$	0.662	0.628
0, 1, 3	0, 1, 2	$\sigma = \left(\frac{1}{2}\right)^5, k = 35$	0.690	0.654
0, 1, 2	1, 2, 3	$\sigma = \left(\frac{1}{2}\right)^4, k = 45$	0.705	0.672
0, 1, 2	0, 2, 3	$\sigma = \left(\frac{1}{2}\right)^3, k = 45$	0.737	0.698
0, 1, 2	0, 1, 3	$\sigma = \left(\frac{1}{2}\right)^3, k = 45$	0.659	0.636
0, 1, 2	0, 1, 2	$\sigma = \left(\frac{1}{2}\right)^3, k = 35$	0.688	0.661

Table S2: Hyperparameter selection results for Gaussian Betti curves, starting at frame 350, labeled by training trajectories.

Glycosylated Trajectories	Aglycosylated Trajectories	Best Hyperparameters	Best Mean Accuracy	Worst Mean Accuracy
1, 2, 3	1, 2, 3	$\sigma = \left(\frac{1}{2}\right)^3, k = 35$	0.707	0.679
1, 2, 3	0, 2, 3	$\sigma = \left(\frac{1}{2}\right)^5, k = 45$	0.732	0.696
1, 2, 3	0, 1, 3	$\sigma = \left(\frac{1}{2}\right)^5, k = 45$	0.645	0.623
1, 2, 3	0, 1, 2	$\sigma = \left(\frac{1}{2}\right)^5, k = 35$	0.674	0.652
0, 2, 3	1, 2, 3	$\sigma = \left(\frac{1}{2}\right)^3, k = 35$	0.775	0.755
0, 2, 3	0, 2, 3	$\sigma = \left(\frac{1}{2}\right)^2, k = 35$	0.795	0.772
0, 2, 3	0, 1, 3	$\sigma = \left(\frac{1}{2}\right)^2, k = 45$	0.737	0.713
0, 2, 3	0, 1, 2	$\sigma = \left(\frac{1}{2}\right)^2, k = 45$	0.756	0.743
0, 1, 3	1, 2, 3	$\sigma = \left(\frac{1}{2}\right)^3, k = 45$	0.717	0.686
0, 1, 3	0, 2, 3	$\sigma = \left(\frac{1}{2}\right)^4, k = 45$	0.741	0.700
0, 1, 3	0, 1, 3	$\sigma = \left(\frac{1}{2}\right)^5, k = 45$	0.657	0.621
0, 1, 3	0, 1, 2	$\sigma = \left(\frac{1}{2}\right)^5, k = 45$	0.682	0.651
0, 1, 2	1, 2, 3	$\sigma = \left(\frac{1}{2}\right)^3, k = 35$	0.705	0.678
0, 1, 2	0, 2, 3	$\sigma = \left(\frac{1}{2}\right)^4, k = 45$	0.728	0.686
0, 1, 2	0, 1, 3	$\sigma = \left(\frac{1}{2}\right)^4, k = 45$	0.649	0.624
0, 1, 2	0, 1, 2	$\sigma = \left(\frac{1}{2}\right)^4, k = 45$	0.672	0.653

Table S3: Hyperparameter selection results for Gaussian Betti curves, starting at frame 500, labeled by training trajectories.

Glycosylated Trajectories	Aglycosylated Trajectories	Best Hyperparameters	Best Mean Accuracy	Worst Mean Accuracy
1, 2, 3	1, 2, 3	$\sigma = \left(\frac{1}{2}\right)^4$, $k = 45$	0.687	0.655
1, 2, 3	0, 2, 3	$\sigma = \left(\frac{1}{2}\right)^3$, $k = 35$	0.706	0.663
1, 2, 3	0, 1, 3	$\sigma = \left(\frac{1}{2}\right)^5$, $k = 45$	0.630	0.604
1, 2, 3	0, 1, 2	$\sigma = \left(\frac{1}{2}\right)^4$, $k = 45$	0.652	0.627
0, 2, 3	1, 2, 3	$\sigma = \left(\frac{1}{2}\right)^3$, $k = 35$	0.772	0.755
0, 2, 3	0, 2, 3	$\sigma = \left(\frac{1}{2}\right)^2$, $k = 35$	0.790	0.766
0, 2, 3	0, 1, 3	$\sigma = \left(\frac{1}{2}\right)^2$, $k = 45$	0.744	0.716
0, 2, 3	0, 1, 2	$\sigma = \left(\frac{1}{2}\right)^2$, $k = 35$	0.755	0.741
0, 1, 3	1, 2, 3	$\sigma = \left(\frac{1}{2}\right)^4$, $k = 45$	0.717	0.673
0, 1, 3	0, 2, 3	$\sigma = \left(\frac{1}{2}\right)^5$, $k = 45$	0.735	0.685
0, 1, 3	0, 1, 3	$\sigma = \left(\frac{1}{2}\right)^5$, $k = 45$	0.658	0.612
0, 1, 3	0, 1, 2	$\sigma = \left(\frac{1}{2}\right)^6$, $k = 35$	0.679	0.635
0, 1, 2	1, 2, 3	$\sigma = \left(\frac{1}{2}\right)^4$, $k = 45$	0.689	0.645
0, 1, 2	0, 2, 3	$\sigma = \left(\frac{1}{2}\right)^3$, $k = 45$	0.706	0.646
0, 1, 2	0, 1, 3	$\sigma = \left(\frac{1}{2}\right)^6$, $k = 45$	0.636	0.593
0, 1, 2	0, 1, 2	$\sigma = \left(\frac{1}{2}\right)^4$, $k = 45$	0.657	0.618

Table S4: Hyperparameter selection results for concatenated normalized 0D, 1D, and 2D Gaussian Betti curves, starting at frame 200, labeled by training trajectories.

Glycosylated Trajectories	Aglycosylated Trajectories	Best Hyperparameters	Best Mean Accuracy	Worst Mean Accuracy
1, 2, 3	1, 2, 3	$\sigma = \left(\frac{1}{2}\right)^1$, $k = 45$	0.672	0.630
1, 2, 3	0, 2, 3	$\sigma = \left(\frac{1}{2}\right)^3$, $k = 35$	0.703	0.664
1, 2, 3	0, 1, 3	$\sigma = \left(\frac{1}{2}\right)^1$, $k = 35$	0.636	0.599
1, 2, 3	0, 1, 2	$\sigma = \left(\frac{1}{2}\right)^3$, $k = 45$	0.657	0.620
0, 2, 3	1, 2, 3	$\sigma = \left(\frac{1}{2}\right)^1$, $k = 35$	0.704	0.643
0, 2, 3	0, 2, 3	$\sigma = \left(\frac{1}{2}\right)^1$, $k = 45$	0.724	0.666
0, 2, 3	0, 1, 3	$\sigma = \left(\frac{1}{2}\right)^1$, $k = 35$	0.671	0.612
0, 2, 3	0, 1, 2	$\sigma = \left(\frac{1}{2}\right)^1$, $k = 35$	0.685	0.634
0, 1, 3	1, 2, 3	$\sigma = \left(\frac{1}{2}\right)^1$, $k = 45$	0.678	0.634
0, 1, 3	0, 2, 3	$\sigma = \left(\frac{1}{2}\right)^1$, $k = 45$	0.705	0.660
0, 1, 3	0, 1, 3	$\sigma = \left(\frac{1}{2}\right)^1$, $k = 45$	0.642	0.602
0, 1, 3	0, 1, 2	$\sigma = \left(\frac{1}{2}\right)^1$, $k = 45$	0.653	0.617
0, 1, 2	1, 2, 3	$\sigma = \left(\frac{1}{2}\right)^1$, $k = 35$	0.677	0.639
0, 1, 2	0, 2, 3	$\sigma = \left(\frac{1}{2}\right)^1$, $k = 45$	0.701	0.661
0, 1, 2	0, 1, 3	$\sigma = \left(\frac{1}{2}\right)^1$, $k = 35$	0.643	0.609
0, 1, 2	0, 1, 2	$\sigma = \left(\frac{1}{2}\right)^1$, $k = 35$	0.655	0.626

Table S5: Hyperparameter selection results for concatenated normalized 0D, 1D, and 2D Gaussian Betti curves, starting at frame 350, labeled by training trajectories.

Glycosylated Trajectories	Aglycosylated Trajectories	Best Hyperparameters	Best Mean Accuracy	Worst Mean Accuracy
1, 2, 3	1, 2, 3	$\sigma = \left(\frac{1}{2}\right)^1$, $k = 45$	0.668	0.627
1, 2, 3	0, 2, 3	$\sigma = \left(\frac{1}{2}\right)^1$, $k = 35$	0.703	0.664
1, 2, 3	0, 1, 3	$\sigma = \left(\frac{1}{2}\right)^1$, $k = 25$	0.634	0.591
1, 2, 3	0, 1, 2	$\sigma = \left(\frac{1}{2}\right)^1$, $k = 45$	0.649	0.621
0, 2, 3	1, 2, 3	$\sigma = \left(\frac{1}{2}\right)^1$, $k = 35$	0.720	0.649
0, 2, 3	0, 2, 3	$\sigma = \left(\frac{1}{2}\right)^1$, $k = 15$	0.745	0.679
0, 2, 3	0, 1, 3	$\sigma = \left(\frac{1}{2}\right)^1$, $k = 35$	0.686	0.615
0, 2, 3	0, 1, 2	$\sigma = \left(\frac{1}{2}\right)^1$, $k = 35$	0.706	0.642
0, 1, 3	1, 2, 3	$\sigma = \left(\frac{1}{2}\right)^1$, $k = 45$	0.674	0.626
0, 1, 3	0, 2, 3	$\sigma = \left(\frac{1}{2}\right)^1$, $k = 15$	0.713	0.658
0, 1, 3	0, 1, 3	$\sigma = \left(\frac{1}{2}\right)^1$, $k = 35$	0.637	0.590
0, 1, 3	0, 1, 2	$\sigma = \left(\frac{1}{2}\right)^1$, $k = 45$	0.650	0.610
0, 1, 2	1, 2, 3	$\sigma = \left(\frac{1}{2}\right)^1$, $k = 45$	0.667	0.630
0, 1, 2	0, 2, 3	$\sigma = \left(\frac{1}{2}\right)^1$, $k = 35$	0.703	0.662
0, 1, 2	0, 1, 3	$\sigma = \left(\frac{1}{2}\right)^1$, $k = 35$	0.634	0.600
0, 1, 2	0, 1, 2	$\sigma = \left(\frac{1}{2}\right)^1$, $k = 45$	0.646	0.617

Table S6: Hyperparameter selection results for concatenated normalized 0D, 1D, and 2D Gaussian Betti curves, starting at frame 500, labeled by training trajectories.

Glycosylated Trajectories	Aglycosylated Trajectories	Best Hyperparameters	Best Mean Accuracy	Worst Mean Accuracy
1, 2, 3	1, 2, 3	$\sigma = \left(\frac{1}{2}\right)^1$, $k = 45$	0.645	0.608
1, 2, 3	0, 2, 3	$\sigma = \left(\frac{1}{2}\right)^1$, $k = 25$	0.692	0.655
1, 2, 3	0, 1, 3	$\sigma = \left(\frac{1}{2}\right)^1$, $k = 15$	0.613	0.574
1, 2, 3	0, 1, 2	$\sigma = \left(\frac{1}{2}\right)^1$, $k = 15$	0.630	0.601
0, 2, 3	1, 2, 3	$\sigma = \left(\frac{1}{2}\right)^1$, $k = 45$	0.714	0.639
0, 2, 3	0, 2, 3	$\sigma = \left(\frac{1}{2}\right)^1$, $k = 45$	0.746	0.669
0, 2, 3	0, 1, 3	$\sigma = \left(\frac{1}{2}\right)^1$, $k = 45$	0.681	0.602
0, 2, 3	0, 1, 2	$\sigma = \left(\frac{1}{2}\right)^1$, $k = 45$	0.692	0.623
0, 1, 3	1, 2, 3	$\sigma = \left(\frac{1}{2}\right)^1$, $k = 45$	0.654	0.619
0, 1, 3	0, 2, 3	$\sigma = \left(\frac{1}{2}\right)^1$, $k = 35$	0.699	0.659
0, 1, 3	0, 1, 3	$\sigma = \left(\frac{1}{2}\right)^1$, $k = 35$	0.618	0.584
0, 1, 3	0, 1, 2	$\sigma = \left(\frac{1}{2}\right)^1$, $k = 45$	0.632	0.601
0, 1, 2	1, 2, 3	$\sigma = \left(\frac{1}{2}\right)^3$, $k = 45$	0.633	0.606
0, 1, 2	0, 2, 3	$\sigma = \left(\frac{1}{2}\right)^1$, $k = 25$	0.679	0.640
0, 1, 2	0, 1, 3	$\sigma = \left(\frac{1}{2}\right)^3$, $k = 45$	0.599	0.573
0, 1, 2	0, 1, 2	$\sigma = \left(\frac{1}{2}\right)^4$, $k = 35$	0.613	0.591

Table S7: Hyperparameter selection results for GCCD matrices, starting at frame 200, labeled by training trajectories.

Glycosylated Trajectories	Aglycosylated Trajectories	Best Hyperparameters	Best Mean Accuracy	Worst Mean Accuracy
1, 2, 3	1, 2, 3	$\sigma = \left(\frac{1}{2}\right)^5, k = 35$	0.912	0.907
1, 2, 3	0, 2, 3	$\sigma = \left(\frac{1}{2}\right)^1, k = 25$	0.944	0.940
1, 2, 3	0, 1, 3	$\sigma = \left(\frac{1}{2}\right)^5, k = 45$	0.916	0.907
1, 2, 3	0, 1, 2	$\sigma = \left(\frac{1}{2}\right)^5, k = 35$	0.913	0.908
0, 2, 3	1, 2, 3	$\sigma = \left(\frac{1}{2}\right)^2, k = 15$	0.913	0.910
0, 2, 3	0, 2, 3	$\sigma = \left(\frac{1}{2}\right)^2, k = 25$	0.940	0.934
0, 2, 3	0, 1, 3	$\sigma = \left(\frac{1}{2}\right)^4, k = 45$	0.915	0.908
0, 2, 3	0, 1, 2	$\sigma = \left(\frac{1}{2}\right)^3, k = 15$	0.909	0.902
0, 1, 3	1, 2, 3	$\sigma = \left(\frac{1}{2}\right)^1, k = 15$	0.908	0.904
0, 1, 3	0, 2, 3	$\sigma = \left(\frac{1}{2}\right)^2, k = 15$	0.934	0.927
0, 1, 3	0, 1, 3	$\sigma = \left(\frac{1}{2}\right)^4, k = 35$	0.907	0.902
0, 1, 3	0, 1, 2	$\sigma = \left(\frac{1}{2}\right)^2, k = 15$	0.899	0.895
0, 1, 2	1, 2, 3	$\sigma = \left(\frac{1}{2}\right)^5, k = 35$	0.917	0.912
0, 1, 2	0, 2, 3	$\sigma = \left(\frac{1}{2}\right)^3, k = 25$	0.943	0.937
0, 1, 2	0, 1, 3	$\sigma = \left(\frac{1}{2}\right)^4, k = 35$	0.915	0.905
0, 1, 2	0, 1, 2	$\sigma = \left(\frac{1}{2}\right)^3, k = 45$	0.908	0.900

Table S8: Hyperparameter selection results for GCCD matrices, starting at frame 350, labeled by training trajectories.

Glycosylated Trajectories	Aglycosylated Trajectories	Best Hyperparameters	Best Mean Accuracy	Worst Mean Accuracy
1, 2, 3	1, 2, 3	$\sigma = \left(\frac{1}{2}\right)^1$, $k = 15$	0.939	0.934
1, 2, 3	0, 2, 3	$\sigma = \left(\frac{1}{2}\right)^1$, $k = 15$	0.932	0.928
1, 2, 3	0, 1, 3	$\sigma = \left(\frac{1}{2}\right)^6$, $k = 25$	0.935	0.928
1, 2, 3	0, 1, 2	$\sigma = \left(\frac{1}{2}\right)^3$, $k = 45$	0.933	0.929
0, 2, 3	1, 2, 3	$\sigma = \left(\frac{1}{2}\right)^1$, $k = 15$	0.941	0.936
0, 2, 3	0, 2, 3	$\sigma = \left(\frac{1}{2}\right)^5$, $k = 25$	0.928	0.921
0, 2, 3	0, 1, 3	$\sigma = \left(\frac{1}{2}\right)^6$, $k = 45$	0.932	0.921
0, 2, 3	0, 1, 2	$\sigma = \left(\frac{1}{2}\right)^4$, $k = 15$	0.928	0.919
0, 1, 3	1, 2, 3	$\sigma = \left(\frac{1}{2}\right)^1$, $k = 15$	0.936	0.929
0, 1, 3	0, 2, 3	$\sigma = \left(\frac{1}{2}\right)^2$, $k = 15$	0.918	0.911
0, 1, 3	0, 1, 3	$\sigma = \left(\frac{1}{2}\right)^4$, $k = 45$	0.922	0.916
0, 1, 3	0, 1, 2	$\sigma = \left(\frac{1}{2}\right)^1$, $k = 25$	0.921	0.916
0, 1, 2	1, 2, 3	$\sigma = \left(\frac{1}{2}\right)^1$, $k = 15$	0.943	0.938
0, 1, 2	0, 2, 3	$\sigma = \left(\frac{1}{2}\right)^3$, $k = 25$	0.927	0.921
0, 1, 2	0, 1, 3	$\sigma = \left(\frac{1}{2}\right)^4$, $k = 35$	0.924	0.914
0, 1, 2	0, 1, 2	$\sigma = \left(\frac{1}{2}\right)^4$, $k = 35$	0.925	0.919

Table S9: Hyperparameter selection results for GCCD matrices, starting at frame 500, labeled by training trajectories.

Glycosylated Trajectories	Aglycosylated Trajectories	Best Hyperparameters	Best Mean Accuracy	Worst Mean Accuracy
1, 2, 3	1, 2, 3	$\sigma = \left(\frac{1}{2}\right)^5$, $k = 35$	0.945	0.940
1, 2, 3	0, 2, 3	$\sigma = \left(\frac{1}{2}\right)^5$, $k = 15$	0.945	0.936
1, 2, 3	0, 1, 3	$\sigma = \left(\frac{1}{2}\right)^5$, $k = 35$	0.944	0.932
1, 2, 3	0, 1, 2	$\sigma = \left(\frac{1}{2}\right)^6$, $k = 45$	0.941	0.930
0, 2, 3	1, 2, 3	$\sigma = \left(\frac{1}{2}\right)^4$, $k = 25$	0.949	0.943
0, 2, 3	0, 2, 3	$\sigma = \left(\frac{1}{2}\right)^3$, $k = 15$	0.942	0.930
0, 2, 3	0, 1, 3	$\sigma = \left(\frac{1}{2}\right)^6$, $k = 25$	0.942	0.930
0, 2, 3	0, 1, 2	$\sigma = \left(\frac{1}{2}\right)^5$, $k = 45$	0.935	0.926
0, 1, 3	1, 2, 3	$\sigma = \left(\frac{1}{2}\right)^1$, $k = 45$	0.954	0.947
0, 1, 3	0, 2, 3	$\sigma = \left(\frac{1}{2}\right)^3$, $k = 15$	0.944	0.937
0, 1, 3	0, 1, 3	$\sigma = \left(\frac{1}{2}\right)^4$, $k = 35$	0.948	0.936
0, 1, 3	0, 1, 2	$\sigma = \left(\frac{1}{2}\right)^6$, $k = 45$	0.946	0.937
0, 1, 2	1, 2, 3	$\sigma = \left(\frac{1}{2}\right)^2$, $k = 25$	0.953	0.949
0, 1, 2	0, 2, 3	$\sigma = \left(\frac{1}{2}\right)^2$, $k = 25$	0.946	0.937
0, 1, 2	0, 1, 3	$\sigma = \left(\frac{1}{2}\right)^5$, $k = 45$	0.946	0.935
0, 1, 2	0, 1, 2	$\sigma = \left(\frac{1}{2}\right)^5$, $k = 45$	0.945	0.934

Table S10: Test accuracies for Gaussian Betti curves, starting at frame 200.

Glycosylated Test Trajectory	Aglycosylated Test Trajectory	Mean Test Accuracy
0	0	0.733
0	1	0.642
0	2	0.853
0	3	0.762
1	0	0.661
1	1	0.588
1	2	0.721
1	3	0.659
2	0	0.704
2	1	0.628
2	2	0.830
2	3	0.748
3	0	0.698
3	1	0.669
3	2	0.812
3	3	0.738
Overall Mean		0.715

Table S11: Test accuracies for Gaussian Betti curves, starting at frame 350.

Glycosylated Test Trajectory	Aglycosylated Test Trajectory	Mean Test Accuracy
0	0	0.709
0	1	0.655
0	2	0.870
0	3	0.786
1	0	0.617
1	1	0.578
1	2	0.669
1	3	0.612
2	0	0.689
2	1	0.646
2	2	0.843
2	3	0.791
3	0	0.702
3	1	0.681
3	2	0.839
3	3	0.780
Overall Mean		0.717

Table S12: Test accuracies for Gaussian Betti curves, starting at frame 500.

Glycosylated Test Trajectory	Aglycosylated Test Trajectory	Mean Test Accuracy
0	0	0.714
0	1	0.708
0	2	0.866
0	3	0.794
1	0	0.599
1	1	0.545
1	2	0.618
1	3	0.585
2	0	0.661
2	1	0.628
2	2	0.817
2	3	0.762
3	0	0.688
3	1	0.695
3	2	0.813
3	3	0.777
Overall Mean		0.704

Table S13: Test accuracies for concatenated normalized 0D, 1D, and 2D Gaussian Betti curves, starting at frame 200.

Glycosylated Test Trajectory	Aglycosylated Test Trajectory	Mean Test Accuracy
0	0	0.704
0	1	0.597
0	2	0.778
0	3	0.698
1	0	0.636
1	1	0.583
1	2	0.681
1	3	0.649
2	0	0.703
2	1	0.614
2	2	0.766
2	3	0.744
3	0	0.698
3	1	0.646
3	2	0.741
3	3	0.728
Overall Mean		0.685

Table S14: Test accuracies for concatenated normalized 0D, 1D, and 2D Gaussian Betti curves, starting at frame 350.

Glycosylated Test Trajectory	Aglycosylated Test Trajectory	Mean Test Accuracy
0	0	0.705
0	1	0.623
0	2	0.762
0	3	0.730
1	0	0.618
1	1	0.569
1	2	0.684
1	3	0.640
2	0	0.721
2	1	0.627
2	2	0.783
2	3	0.763
3	0	0.728
3	1	0.644
3	2	0.767
3	3	0.758
Overall Mean		0.695

Table S15: Test accuracies for concatenated normalized 0D, 1D, and 2D Gaussian Betti curves, starting at frame 500.

Glycosylated Test Trajectory	Aglycosylated Test Trajectory	Mean Test Accuracy
0	0	0.704
0	1	0.613
0	2	0.727
0	3	0.696
1	0	0.589
1	1	0.505
1	2	0.635
1	3	0.598
2	0	0.709
2	1	0.601
2	2	0.759
2	3	0.745
3	0	0.687
3	1	0.636
3	2	0.745
3	3	0.697
Overall Mean		0.665

Table S16: Test accuracies for GCCD matrices, starting at frame 200.

Glycosylated Test Trajectory	Aglycosylated Test Trajectory	Mean Test Accuracy
0	0	0.937
0	1	0.851
0	2	0.959
0	3	0.942
1	0	0.917
1	1	0.850
1	2	0.958
1	3	0.948
2	0	0.930
2	1	0.864
2	2	0.993
2	3	0.966
3	0	0.932
3	1	0.879
3	2	0.989
3	3	0.981
Overall Mean		0.931

Table S17: Test accuracies for GCCD matrices, starting at frame 350.

Glycosylated Test Trajectory	Aglycosylated Test Trajectory	Mean Test Accuracy
0	0	0.928
0	1	0.901
0	2	0.927
0	3	0.930
1	0	0.903
1	1	0.923
1	2	0.949
1	3	0.942
2	0	0.936
2	1	0.956
2	2	0.995
2	3	0.981
3	0	0.936
3	1	0.957
3	2	0.978
3	3	0.988
Overall Mean		0.946

Table S18: Test accuracies for GCCD matrices, starting at frame 500.

Glycosylated Test Trajectory	Aglycosylated Test Trajectory	Mean Test Accuracy
0	0	0.975
0	1	0.957
0	2	0.960
0	3	0.967
1	0	0.940
1	1	0.930
1	2	0.966
1	3	0.967
2	0	0.950
2	1	0.951
2	2	0.989
2	3	0.987
3	0	0.958
3	1	0.964
3	2	0.998
3	3	0.996
Overall Mean		0.966

Table S19: Fc Domain GCCD matrix run times in seconds (s) in Julia.

Dataset	Traj.	Frame	Run 0	Run 1	Run 2	Run 3	Run 4	Run 5	Run 6
Glycosylated	3	955	19.9	20.4	18.3	21.6	19.0	20.3	20.6
Aglycosylated	3	809	21.9	20.8	19.1	21.9	20.1	20.3	20.5
Aglycosylated	0	445	21.8	20.2	18.6	21.6	19.3	20.1	21.0
Glycosylated	0	531	19.9	21.2	18.8	21.6	19.2	18.9	20.8
Glycosylated	2	721	19.0	20.2	19.2	21.3	18.8	18.9	20.2
Aglycosylated	1	388	19.4	21.3	19.8	21.6	19.8	19.3	20.5
Aglycosylated	0	980	20.5	21.4	18.8	21.2	19.8	19.0	20.5
Aglycosylated	0	317	19.2	20.5	19.0	22.0	19.2	18.9	20.5
Glycosylated	3	284	19.6	21.9	19.2	22.1	19.5	19.3	21.4
Aglycosylated	0	904	20.4	21.1	20.2	22.3	19.6	19.8	20.7
Glycosylated	3	733	19.4	20.4	18.7	21.8	19.4	19.5	20.7
Aglycosylated	1	979	20.4	20.8	22.3	22.1	20.3	19.0	21.6
Glycosylated	2	389	20.7	21.1	21.7	19.0	20.2	19.1	20.4
Aglycosylated	1	334	19.9	21.0	22.0	19.6	19.6	19.3	21.0
Glycosylated	1	540	20.2	19.9	22.1	18.6	19.9	19.5	20.5
Glycosylated	2	908	20.3	18.8	21.1	18.8	20.2	19.6	20.4
Glycosylated	1	544	20.2	18.5	22.1	18.9	20.7	20.7	20.7
Aglycosylated	3	791	21.1	19.0	21.5	19.8	20.7	21.1	20.7
Glycosylated	0	976	20.8	19.0	21.8	19.2	20.7	22.0	21.4
Aglycosylated	2	465	20.6	19.2	21.8	19.4	20.1	21.8	21.4

Table S20: Fc Domain GCCD matrix run times in seconds (s) in Python.

Dataset	Traj.	Frame	Run 0	Run 1	Run 2	Run 3	Run 4	Run 5	Run 6
Glycosylated	3	955	38.8	38.9	39.2	38.9	39.0	38.9	39.0
Aglycosylated	3	809	39.6	39.8	39.9	39.8	39.6	39.5	39.6
Aglycosylated	0	445	40.5	40.5	40.1	40.1	40.4	40.4	40.4
Glycosylated	0	531	39.1	39.2	39.2	39.4	39.4	39.5	39.1
Glycosylated	2	721	39.2	39.4	39.3	39.0	39.3	39.1	39.1
Aglycosylated	1	388	39.3	39.3	39.3	39.3	39.1	39.5	39.6
Aglycosylated	0	980	39.5	39.0	39.4	39.5	39.7	39.7	39.5
Aglycosylated	0	317	40.1	40.3	40.5	40.5	40.1	40.0	40.0
Glycosylated	3	284	39.6	39.4	39.5	39.1	39.5	39.5	39.6
Aglycosylated	0	904	40.5	40.5	40.4	40.5	40.4	40.6	40.2
Glycosylated	3	733	40.5	40.5	40.5	40.4	40.2	40.1	40.2
Aglycosylated	1	979	40.4	40.5	40.3	40.0	40.3	40.5	40.7
Glycosylated	2	389	39.4	39.6	39.4	39.5	39.8	39.7	39.4
Aglycosylated	1	334	39.7	39.8	40.0	39.9	39.6	39.4	39.6
Glycosylated	1	540	39.8	39.5	39.2	39.0	39.3	39.4	39.5
Glycosylated	2	908	39.8	39.3	40.4	39.7	39.5	39.9	39.7
Glycosylated	1	544	40.2	40.3	40.6	40.4	40.4	40.3	40.0
Aglycosylated	3	791	40.8	41.1	40.9	40.6	40.7	41.0	41.0
Glycosylated	0	976	38.7	38.8	39.0	39.1	39.1	39.1	38.9
Aglycosylated	2	465	40.9	41.0	41.2	41.1	41.2	41.1	41.2

Table S21: 2HII GCCD matrix run times in seconds (s) in Julia.

Run 0	Run 1	Run 2	Run 3	Run 4	Run 5	Run 6
94.4	94.0	94.1	96.3	92.6	94.9	98.0

Table S22: 2HII GCCD matrix run times in seconds (s) in Python.

Run 0	Run 1	Run 2	Run 3	Run 4	Run 5	Run 6
173.2	166.9	169.2	172.8	167.0	169.5	169.8
